# Functional characterization of adaptive variation within a *cis*-regulatory element influencing *Drosophila melanogaster* growth

**DOI:** 10.1371/journal.pbio.2004538

**Published:** 2018-01-11

**Authors:** Amanda Glaser-Schmitt, John Parsch

**Affiliations:** Faculty of Biology, Ludwig-Maximilians-Universität München, Munich, Germany; The Institute of Science and Technology Austria, Austria

## Abstract

Gene expression variation is a major contributor to phenotypic diversity within species and is thought to play an important role in adaptation. However, examples of adaptive regulatory polymorphism are rare, especially those that have been characterized at both the molecular genetic level and the organismal level. In this study, we perform a functional analysis of the *Drosophila melanogaster CG9509* enhancer, a *cis*-regulatory element that shows evidence of adaptive evolution in populations outside the species’ ancestral range in sub-Saharan Africa. Using site-directed mutagenesis and transgenic reporter gene assays, we determined that 3 single nucleotide polymorphisms are responsible for the difference in *CG9509* expression that is observed between sub-Saharan African and cosmopolitan populations. Interestingly, while 2 of these variants appear to have been the targets of a selective sweep outside of sub-Saharan Africa, the variant with the largest effect on expression remains polymorphic in cosmopolitan populations, suggesting it may be subject to a different mode of selection. To elucidate the function of *CG9509*, we performed a series of functional and tolerance assays on flies in which *CG9509* expression was disrupted. We found that *CG9509* plays a role in larval growth and influences adult body and wing size, as well as wing loading. Furthermore, variation in several of these traits was associated with variation within the *CG9509* enhancer. The effect on growth appears to result from a modulation of active ecdysone levels and expression of growth factors. Taken together, our findings suggest that selection acted on 3 sites within the *CG9509* enhancer to increase *CG9509* expression and, as a result, reduce wing loading as *D*. *melanogaster* expanded out of sub-Saharan Africa.

## Author summary

Much of the phenotypic variation that is observed within species is thought to be caused by variation in gene expression. Variants within *cis*-regulatory elements, which affect the expression of nearby genes within the same DNA strand, are thought to be an abundant resource upon which natural selection can act. Understanding the functional consequences of adaptive *cis*-regulatory changes is important, as it can help elucidate the mechanisms underlying phenotypic evolution in general and provide insight into the development and maintenance of biodiversity. However, functional analyses of these types of changes remain rare. Here we present a functional analysis of an adaptively evolving enhancer element of a *D*. *melanogaster* gene called *CG9509*, of previously unknown function. We show that 3 single nucleotide polymorphisms located within the enhancer of this gene are responsible for an increase in *CG9509* expression in cosmopolitan populations (outside of south and central Africa) relative to sub-Saharan populations, which include ancestral populations. We further show that *CG9509* is involved in the regulation of growth rate and body size determination and propose that the *CG9509* enhancer underwent positive selection to reduce wing loading as the species expanded out of sub-Saharan Africa.

## Introduction

Gene expression variation is extensive both within and between species and is believed to underlie much of the phenotypic diversity observed among species, as well as among populations of the same species [1–2]. Furthermore, expression variation is thought to provide an abundant source of material for adaptation, as alterations in gene expression are more easily fine-tuned on a temporal and tissue-specific scale than changes in protein structure [3–4]. In particular, *cis*-regulatory elements, which are adjacent to genes and directly affect their expression, are thought to be frequent targets of adaptive evolution [2–6]. Despite this prediction, examples of adaptive *cis*-regulatory changes remain comparatively rare, although the number of such examples continues to grow [7–16]. The discrepancy between the predicted abundance and actual instances of identified adaptive *cis*-regulatory divergence is likely in part due to the difficulty in detecting regulatory adaptation, as well as in determining its effect on an organismal phenotype that may be the target of selection. Even in some of the best-studied species, the function of many genes remains unknown, and alterations in those with known functions often have pleiotropic effects, making it difficult to determine the link between an expression change and an adaptive organismal phenotype. As more instances of adaptive *cis*-regulatory evolution are uncovered, it is important to identify the genetic and molecular mechanisms that underlie them, which can help to further our understanding of the mechanisms of phenotypic evolution and shed light on the origins of biodiversity [17]. However, studies performing in-depth functional analyses of individual adaptively evolving *cis-*regulatory elements remain even more rare than those documenting adaptive *cis*-regulatory divergence (e.g., [18–21]).

**Fig 1:**
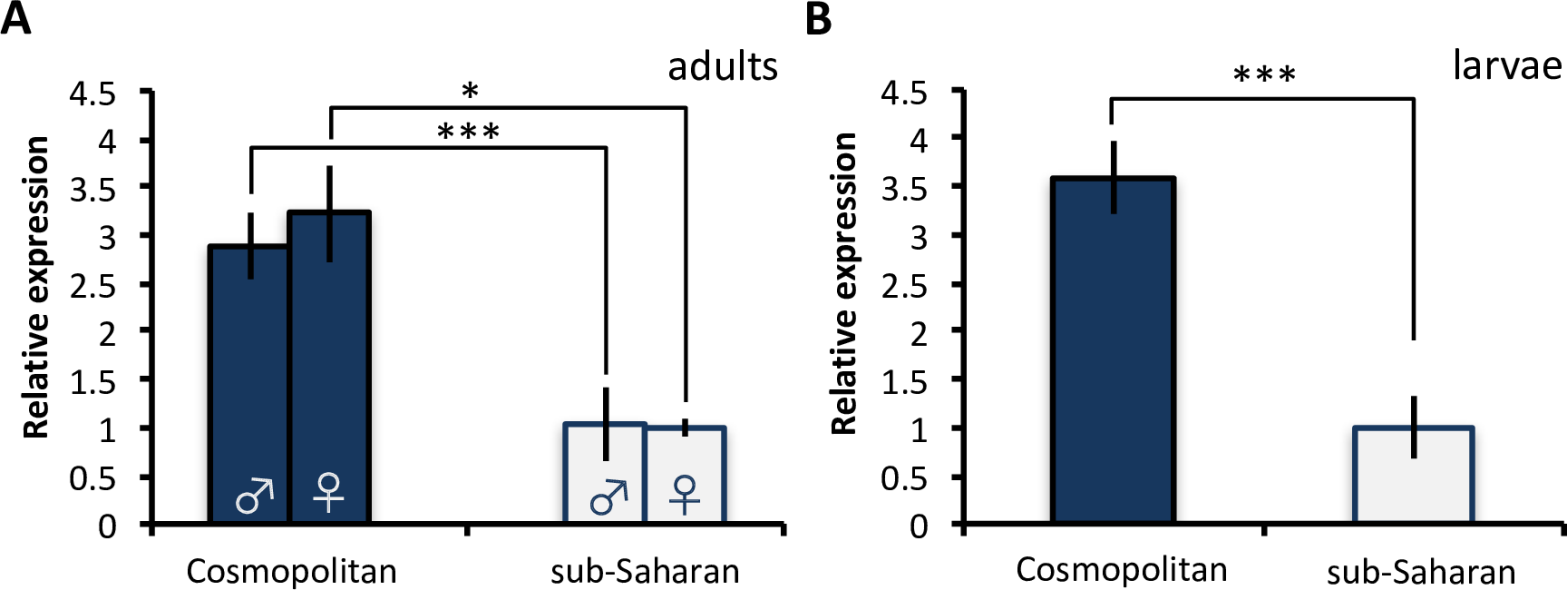
*CG9509* expression in cosmopolitan and sub-Saharan African *D*. *melanogaster*. Relative expression levels as determined by quantitative reverse transcription PCR (qRT-PCR). Shown are (A) adult males (data from [31]) and females (data from [12]) and (B) late wandering third instar larvae (*N* = 21–35 isofemale strains per type with 2 biological replicates per strain). Blue bars represent cosmopolitan flies, and white bars represent sub-Saharan flies. Underlying data can be found in S1 Data. Error bars show the standard error of the mean. Differences between populations were assessed with a *t* test. *P < 0.05, ***P < 0.005.

**Fig 2:**
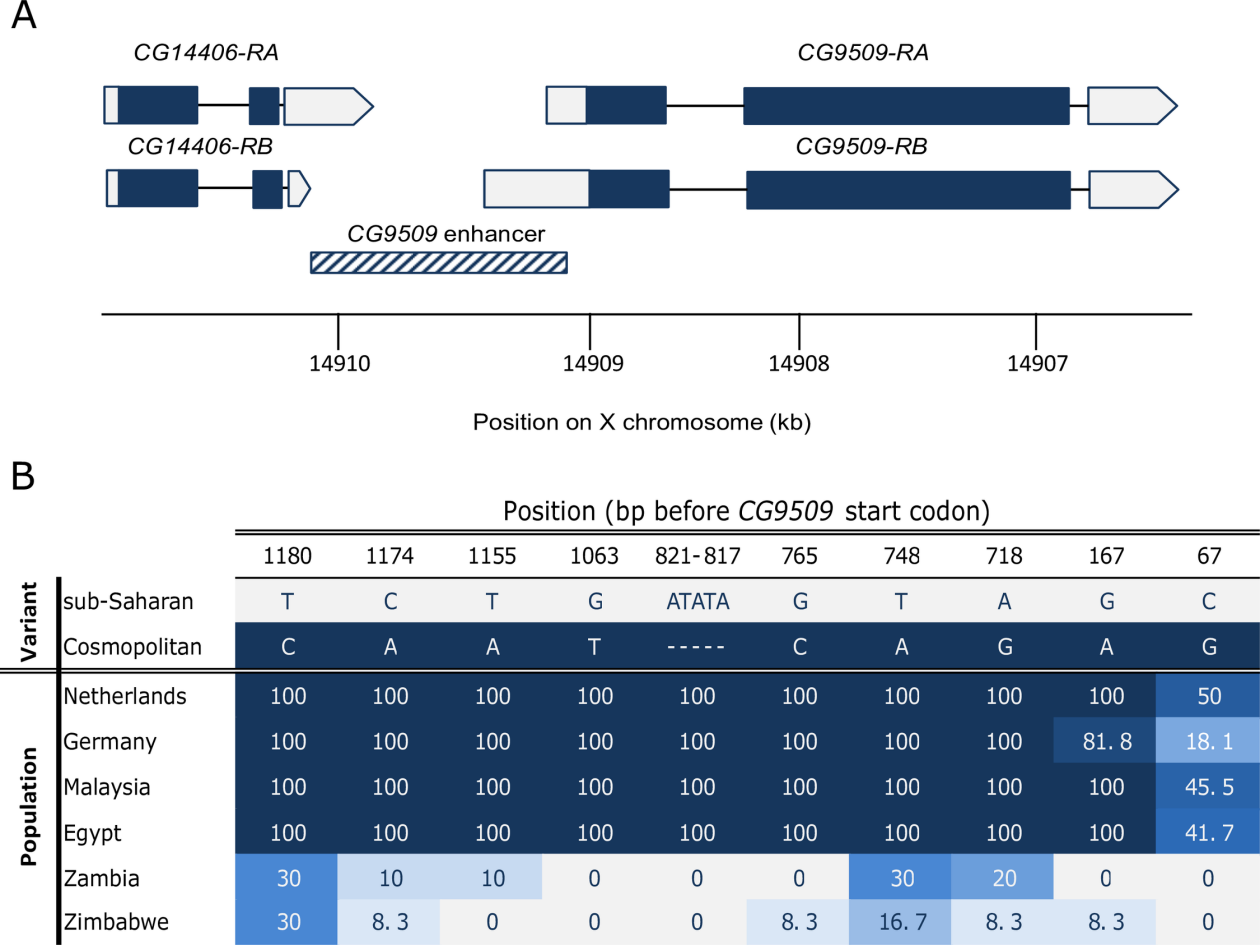
The *CG9509* gene region. (A) Schematic of the *CG9509* gene and enhancer. Blue boxes represent exons, and white boxes represent untranslated regions, with the pointed ends indicating the direction of transcription. RNA sequencing (RNA-seq) data [28] indicate that the *CG9509*-*RA* transcript is more abundant, accounting for >99% of all transcripts. The *CG9509* enhancer is indicated by a hatched box. (B) Single nucleotide polymorphisms (SNPs) and indels in the *CG9509* enhancer with >10% frequency in cosmopolitan populations. Cosmopolitan sequence variants are indicated in blue, and sub-Saharan variants are indicated in white, with lighter shades of blue indicating a mixture of both variants in the population. For each population, the observed frequency of the cosmopolitan variant (in percent) is shown [31].

Transcriptomic methods have proven effective at identifying putatively adaptive alterations in gene expression within and between species [22–28]. *CG9509* is a gene initially identified as a candidate for adaptive *cis*-regulatory divergence through one such study that compared expression between a derived, European and an ancestral, sub-Saharan African (henceforth sub-Saharan) population of *D*. *melanogaster* [24]. Until now, the function of *CG9509* has remained unknown, although it has been predicted to have oxidoreductase activity [29] and/or play a role in ecdysteroid metabolism [30]. Adult *CG9509* expression was found to be 2–3-fold higher in the European population than in the sub-Saharan population (Fig 1A) [12,24], and this expression difference extends to other cosmopolitan (here defined as populations outside of south and central Africa) and sub-Saharan populations [31]. Transgenic reporter gene experiments revealed that variation within a 1.2-kb *cis*-regulatory element upstream of the gene (referred to here as the *CG9509* enhancer, Fig 2A) can account fully for the expression divergence and shows evidence of a selective sweep in cosmopolitan populations [12,31]. This suggests that positive selection acted on the *CG9509* enhancer to increase *CG9509* expression after *D*. *melanogaster*’s expansion out of sub-Saharan Africa, which is estimated to have occurred approximately 15,000 years ago [32–34], but before the separation of European and Asian populations approximately 2,500–5,000 years ago [31,35]. Within the *CG9509* enhancer, there are 9 single nucleotide polymorphisms (SNPs) and 1 insertion/deletion (indel) polymorphism (Fig 2B) that show large frequency differences between the populations and are candidates for the target(s) of selection responsible for the expression divergence. In all cases, the cosmopolitan variant is inferred to be the derived state, while the sub-Saharan variant is inferred to be ancestral [31].

In this study, we use site-directed mutagenesis and transgenic reporter genes to determine the effect of individual SNP and indel variants within the *CG9509* enhancer on gene expression. We also use RNA interference (RNAi) and a newly discovered *CG9509* hypomorph allele to reveal some of *CG9509*’s previously unknown biological functions. We find that 3 SNPs within the *CG9509* enhancer contribute to the expression divergence seen between cosmopolitan and sub-Saharan alleles. Interestingly, 2 SNPs that have a small effect on expression are fixed in cosmopolitan populations and appear to have been the targets of a selective sweep, while the SNP with the largest effect on expression is at intermediate frequency in cosmopolitan populations, suggesting that another type of selection may be acting on this site. We also show that *CG9509* expression influences larval growth and thus plays a role in determining adult body size and wing loading (i.e., the ratio of body mass to wing area). Furthermore, the genetic variants influencing *CG9509* expression are associated with variation in these phenotypic traits. Our results suggest that selection on the 3 SNPs within the *CG9509* enhancer occurred in order to reduce wing loading outside of sub-Saharan Africa.

## Results

### Larval *CG9509* expression divergence

Previous studies focused solely on adult *CG9509* expression variation [12,31]. To determine if the adult expression pattern is established earlier in development, we surveyed larval *CG9509* expression. Because *D*. *melanogaster* developmental gene expression is highly dynamic with a high transcriptional turnover, even among stages that are only a few hours apart [36], we focused our expression analysis on a well-established larval stage in order to ensure that any observed expression divergence is due to population divergence rather than developmental stage variation. To this end, we surveyed *CG9509* expression in late wandering third instar larvae of 3 cosmopolitan populations (the Netherlands, Egypt, and Malaysia) and 2 sub-Saharan populations (Zimbabwe and Zambia). Similar to adults (Fig 1A), larval *CG9509* expression in cosmopolitan populations was significantly higher than in sub-Saharan populations by 3–5.5-fold (*t* test, *P* < 5 × 10^−4^, Fig 1B and S1 Fig).

### Functional analysis of sequence variants in the *CG9509* enhancer

**Fig 3:**
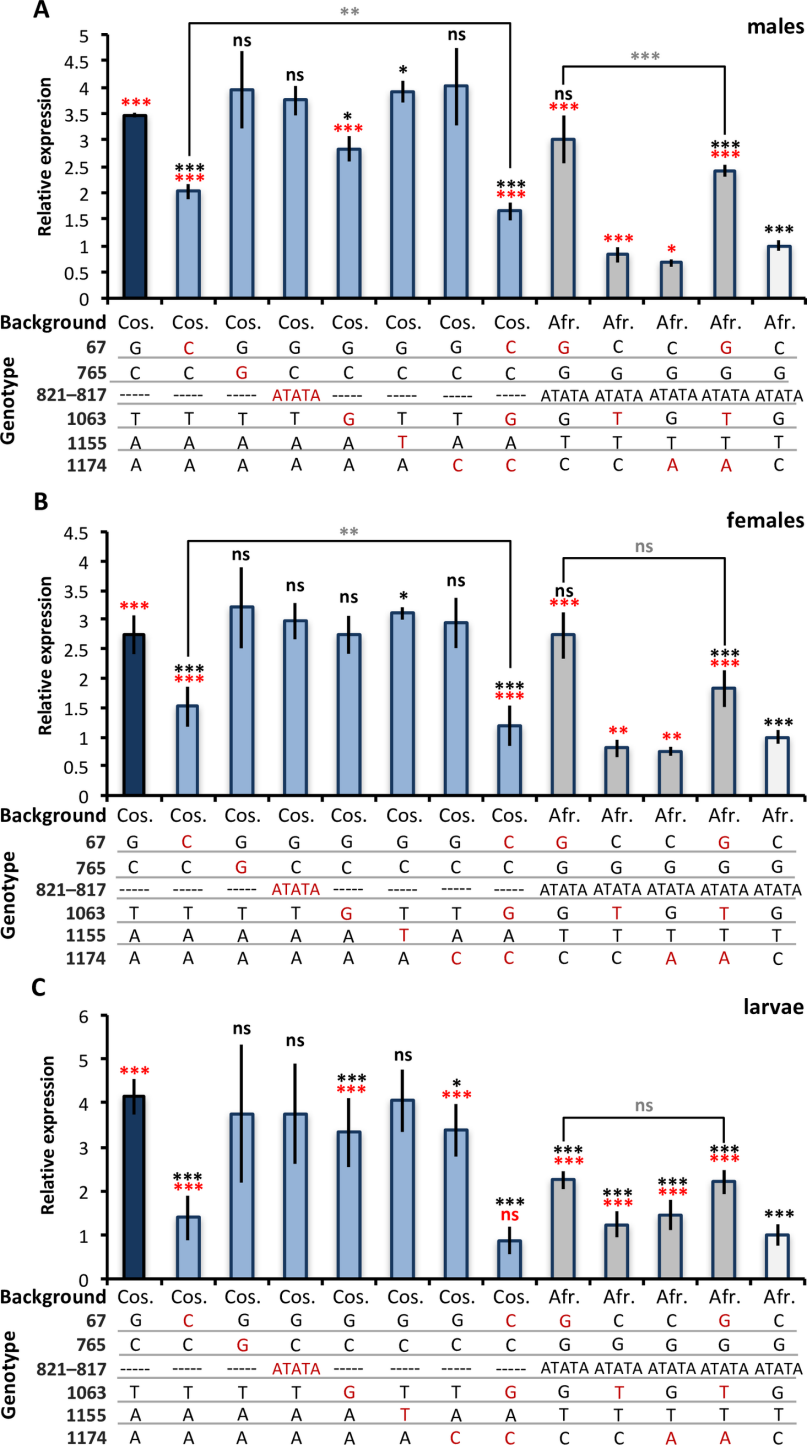
Transgenic reporter gene expression. Reporter gene expression in (A) males, (B) females, and (C) larvae (*N* = 4–8 per strain and sex or stage). Expression of the *LacZ* reporter gene was measured with a β-galactosidase enzymatic assay. Blue bars indicate expression driven by a wild-type cosmopolitan (Cos.) enhancer, while white bars show expression driven by a wild-type sub-Saharan African (Afr.) enhancer. Light blue bars indicate expression driven by the enhancer after mutations were introduced into a cosmopolitan background. Dark gray bars show expression driven by the enhancer after mutations were introduced in a sub-Saharan African background. “Genotype” indicates the nucleotides at positions 1174, 1155, 1063, 821–817, 765, and 67 before the *CG9509* start codon, respectively. Mutated sites are shown in red. Underlying data can be found in S1 Data. Significance was assessed using a *t* test, and a Bonferroni multiple test correction was applied. Significance is represented by black asterisks for comparisons to the original cosmopolitan enhancer, red asterisks for the original sub-Saharan enhancer, and gray asterisks for comparisons between mutated enhancers. ns, not significant; *P < 0.05, **P < 0.01, ***P < 0.005.

To determine which variants in the *CG9509* enhancer (Fig 2B) contribute to the expression divergence between cosmopolitan and sub-Saharan *D*. *melanogaster* (Fig 1 and S1 Fig), we created a series of transgenic reporter gene constructs that were introduced into the *D*. *melanogaster* genome. Because these reporter gene constructs were tested in a common genomic background, our results should be free from the confounding effects of *trans*-acting factors. Briefly, a cosmopolitan and a sub-Saharan enhancer allele were cloned in front of a *LacZ* reporter gene, and 6 sites of interest at positions 1174, 1155, 1063, 821–817, 765, and 67 (Fig 2B) were mutated individually and in various combinations (Fig 3). The tested sites were chosen as those at which the derived variant was fixed in all cosmopolitan populations, but at a frequency ≤10% in sub-Saharan populations. Position 67, at which the derived variant is at intermediate frequency in cosmopolitan populations but absent from sub-Saharan populations (Fig 2B), was also tested because it had previously been associated with *CG9509* expression variation [31,37]. Mutations were first introduced into the cosmopolitan enhancer allele, changing the nucleotide(s) to the ancestral (sub-Saharan) state. Sites found to have an effect on expression in the cosmopolitan background were then mutated to the cosmopolitan state in the sub-Saharan background. Adult and larval expression driven by the cosmopolitan enhancer was 3–4-fold higher than that driven by the sub-Saharan enhancer (Fig 3), which is in line with the expression divergence found in natural populations (Fig 1 and S1 Fig). Thus, the *CG9509* enhancer can account for nearly all of the expression divergence between cosmopolitan and sub-Saharan larvae and adults.

#### One SNP accounts for most of the expression divergence in adults

We determined that the SNP at position 67 (Fig 2B) accounts for the majority of adult expression divergence between cosmopolitan and sub-Saharan populations. The sub-Saharan “C” variant of this SNP is at intermediate frequency in cosmopolitan populations and, when introduced into the cosmopolitan enhancer background, leads to an approximately 45% reduction in expression (Fig 3A and 3B). However, expression was still significantly higher than that driven by the sub-Saharan enhancer (Fig 3A and 3B). When the cosmopolitan “G” variant was introduced into the sub-Saharan background, expression increased 3-fold (Fig 3A and 3B), resulting in expression equivalent to cosmopolitan levels (Fig 3A and 3B). Thus, while the derived variant “G” is sufficient to produce adult cosmopolitan expression in a sub-Saharan background, background-specific epistatic effects, presumably due to other site(s) within the cosmopolitan enhancer, appear to prevent a complete return to sub-Saharan expression when the “C” variant is introduced into a cosmopolitan background.

To better understand how the variant at position 67 affects adult expression, we performed an ANOVA using sex, background, the variant at position 67, and the interaction between the variant at position 67 and background, sex, and the other tested sites within the *CG9509* enhancer. We found that background, sex, and the variant at position 67 had a highly significant effect on expression (*P* < 0.001, S1 Table); however, the interaction between the variant at position 67 and background was not significant (*P* > 0.1, S1 Table). On the other hand, the interaction between the variant at position 67 and sex (*P* < 0.005, S1 Table), as well as 4 of the 5 other tested variants, was significant (*P* < 0.05 for each, S1 Table), suggesting that (1) our inability to recreate sub-Saharan expression in a cosmopolitan background is likely due to epistatic effects with other sites in the *CG9509* enhancer haplotype rather than the genomic background as a whole and (2) epistatic interactions between sites in regulatory regions may be common. Indeed, we detected several sex-, background-, and/or stage-specific effects in our reporter gene assays (Fig 3). Next, we calculated partial eta squared (*η*^*2*^_*p*_), which is a standardized measure of effect size in which effect sizes on the magnitude of approximately 0.01, 0.06, and 0.14 are generally considered to represent small, medium, and large effect sizes, respectively [38]. All of the tested factors had a large effect on expression (*η*^*2*^_*p*_ > 0.12, S1 Table). The largest effect was for the variant at position 67 (*η*^*2*^_*p*_ = 0.975, S1 Table), followed by sex (*η*^*2*^_*p*_ = 0.896, S1 Table) and background (*η*^*2*^_*p*_ = 0.772, S1 Table), suggesting that the variant at position 67 is the strongest predictor of adult *CG9509* expression.

#### Three SNPs contribute to the expression divergence in larvae

We identified 3 SNPs in the *CG9509* enhancer at positions 67, 1063, and 1174 (Fig 2B) that can account for the majority of the expression divergence between cosmopolitan and sub-Saharan African larvae. Of the 3, the SNP at position 67, which accounts for the majority of adult expression divergence, has the largest individual effect on expression (Fig 3). Introduction of the cosmopolitan “G” variant into a sub-Saharan background increased expression by 2.25-fold (Fig 3C), while introduction of the sub-Saharan “C” variant into the cosmopolitan background reduced expression by 2-fold (Fig 3C). At position 1063, introducing the cosmopolitan “T” variant into the sub-Saharan background increased expression by 25% (Fig 3C), while introducing a sub-Saharan “G” variant into the cosmopolitan background reduced expression by 20% (Fig 3C). Similarly, at position 1174, introducing a sub-Saharan “C” variant into the cosmopolitan background reduced expression by 20% (Fig 3C), while introducing a cosmopolitan “A” variant into the sub-Saharan background increased expression by 50% (Fig 3C). When the sub-Saharan variants at all 3 positions were introduced together in the cosmopolitan background, expression was reduced to levels equivalent to sub-Saharan expression (Fig 3C). When the cosmopolitan variants at all 3 positions were introduced together in the sub-Saharan background, expression increased 2.2-fold; however, it remained 50% lower than cosmopolitan expression (Fig 3C). Furthermore, when all 3 cosmopolitan variants were present in the sub-Saharan background, expression did not differ from when only the cosmopolitan “G” variant at position 67 was introduced (*t* test, *P* = 1, Fig 3C). Thus, background-specific epistatic effects, presumably caused by interactions with other sub-Saharan variants within the *CG9509* enhancer, appear to prevent expression from reaching the full cosmopolitan level in a sub-Saharan background. Our expression analyses corroborate these findings. When larval *CG9509* expression in each population is partitioned according to the variant at position 67, the “G” variant makes a large contribution to the expression divergence observed between cosmopolitan and sub-Saharan larvae but cannot account for all of it (S1 Fig).

### Functional analysis of *CG9509*

In order to elucidate the function of *CG9509* and the effects of its expression on organismal phenotypes, we performed a series of functional and tolerance assays on flies in which *CG9509* expression was disrupted. For this, we used both a newly identified hypomorph allele and RNAi. The hypomorph allele (*CG9509*^*del*^) was discovered as a spontaneous mutation in an isofemale line derived from Munich, Germany. It contains a frameshift-causing deletion within the *CG9509* coding region and shows greatly reduced levels of *CG9509* mRNA (S2 Fig). As a control, flies homozygous for *CG9509*^*del*^ were compared to wild-type isofemale lines derived from the same population at the same time. RNAi knockdown of *CG9509* expression was achieved by crossing a ubiquitous *Act5C*-*GAL4* driver line to a transgenic line containing an RNAi hairpin construct specific to *CG9509* (RNAi-*CG9509*) and flanked by a yeast upstream activating sequence (UAS). As a control, *CG9509* knockdown flies were compared to flies of the host strain from which they were derived (UAS^-^).

#### Effect of *CG9509* expression on cold, ethanol, and insecticide tolerance

Flies with disrupted *CG9509* expression were tested for cold, ethanol, and insecticide (DDT and malathion) tolerance. If *CG9509* expression plays a role in tolerance to cold temperatures or any of the tested compounds, we expect that a decrease in *CG9509* expression should lead to a decrease in tolerance. *CG9509* expression was not positively associated with tolerance to cold or the tested compounds and, unexpectedly, was negatively associated with insecticide and female cold tolerance (Table 1, S3 Fig).

**Table 1:** Effect of *CG9509* knockdown in adult tolerance assays.

Tolerance assay	Effect^a^	*P* value^b^
DDT	+	0.013
Malathion	+	2.84 × 10^−5^
Cold (females)	+	0.009
Cold (males)	0	0.248
Ethanol	0	0.733

^a^Directional effect of *CG9509* knockdown on tolerance.

^b^*P* values were obtained using generalized linear models (glms) with a quasibinomial distribution using sex, fly strain, and compound concentration as factors or, for cold tolerance, a *t* test.

#### Effect of *CG9509* expression on developmental timing, larval growth rate, adult body size, and wing loading

**Fig 4:**
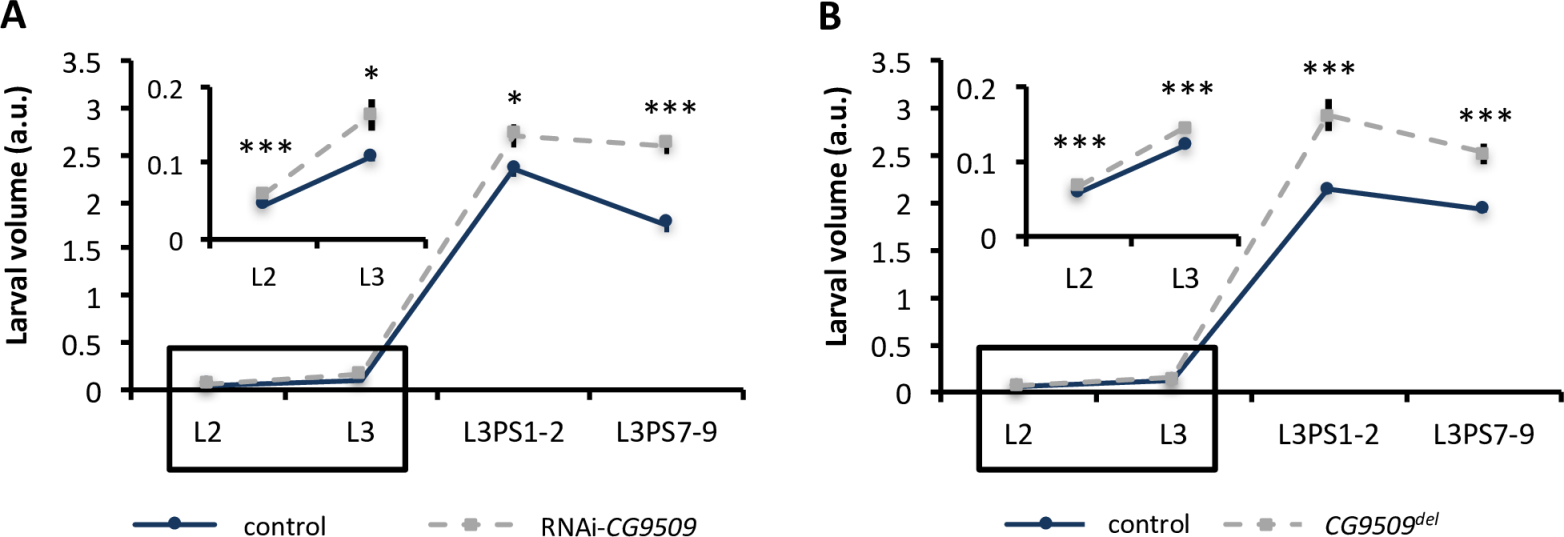
Effect of *CG9509* expression on larval growth rate. Larval volume in arbitrary units (a.u.) in (A) control (blue lines; *N* = 15–19 per stage) and RNAi-*CG9509* (gray, hatched lines; *N* = 15–20 per stage) larvae and (B) control (blue lines; *N* = 60 per stage) and *CG9509*^*del*^ (gray, hatched lines; *N* = 15 per stage) larvae. Four larval stages were examined: second instar larvae (L2) after the first-to-second instar larval molt, third instar larvae (L3) after the second-to-third instar larval molt, early wandering third instar larvae (L3PS1-2), and late wandering third instar larvae (L3PS7-9). Insets show the boxed region on a larger scale. Underlying data can be found in S1 Data. Error bars represent the standard error of the mean. Significance was assessed via a *t* test for each larval stage, and a Bonferroni multiple test correction was applied. *P < 0.05, ***P < 0.005.

**Fig 5:**
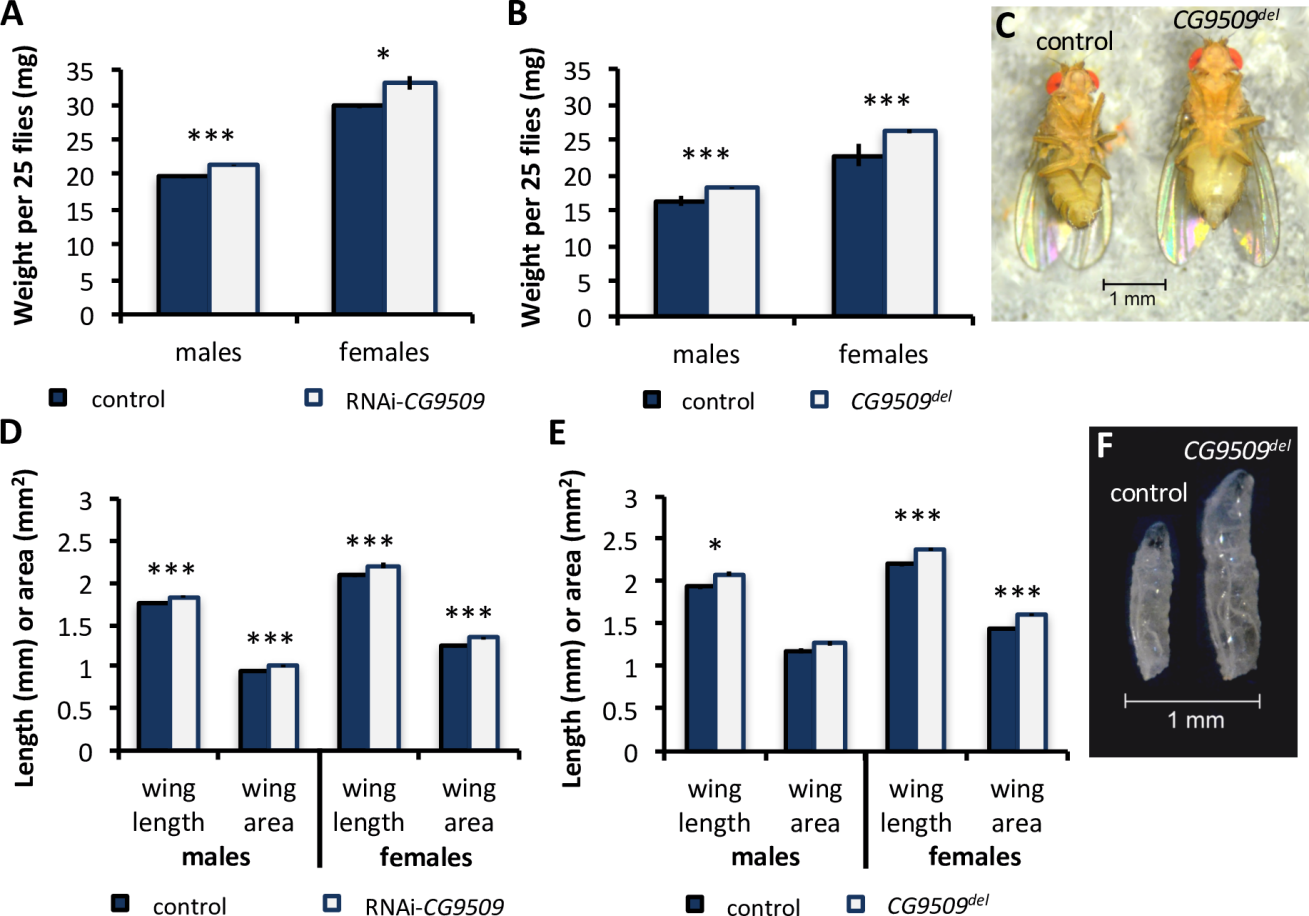
Effect of *CG9509* expression on body size. (A, B) Body weight per 25 flies in (A) control (blue; *N* = 5–6 per sex) and RNAi-*CG9509* (white; *N* = 4 per sex) flies and (B) control (blue; *N* = 16 per sex) and *CG9509*^*del*^ (white; *N* = 6–7 per sex) flies. (D, E) Wing length and wing area in (D) control (blue) and RNAi-*CG9509* (white) flies (*N* = 15 per line and sex) and (E) control (blue; *N* = 20 per sex) and *CG9509*^*del*^ (white; *N* = 9–13 per sex) flies. (C, F) Pictured are control (left) and *CG9509*^*del*^ (C) females and (F) third instar larvae after the second-to-third instar molt. Underlying data can be found in S1 Data. Error bars represent the standard error of the mean. Significance was assessed via *t* test. *P < 0.05, ***P < 0.005.

**Fig 6:**
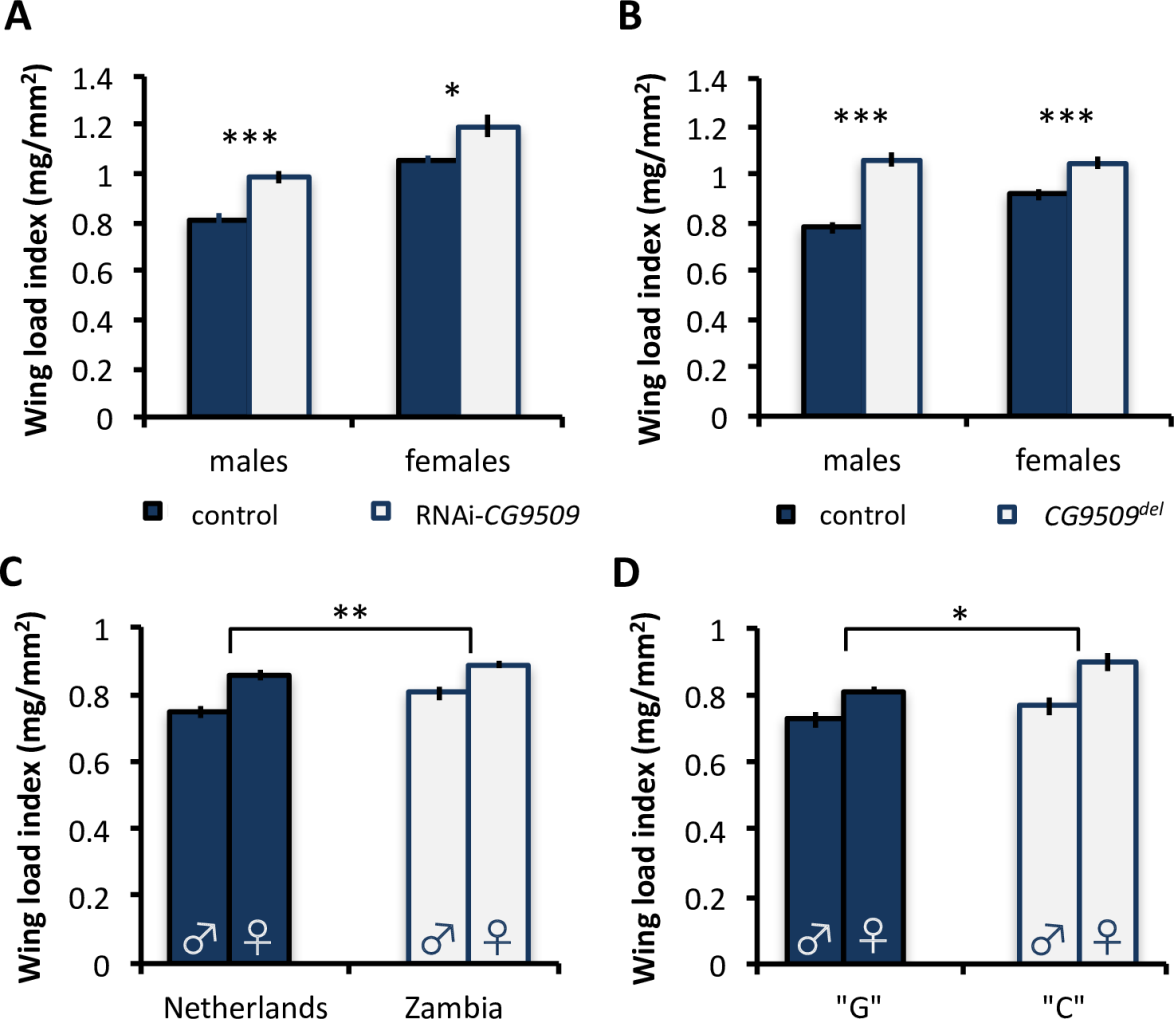
Effect of *CG9509* expression on wing loading. Wing load index in (A) control (blue; *N* = 10–15 per sex) and RNAi-*CG9509* (white; *N* = 10 per sex) flies, (B) control (blue; *N* = 20 per sex) and *CG9509*^*del*^ (white; *N* = 14–15 per sex) flies, (C) flies from a Dutch (blue; *N* = 12 isofemale strains with 5 replicates each per sex) and a Zambian (white; *N* = 10 strains with 5 replicates each per sex) population, and (D) flies from the Dutch population partitioned according to the sequence variant at position 67. The high-expression, cosmopolitan “G” variant (6 strains with 5 replicates each per sex) is shown in blue, and the low*-*expression, sub-Saharan “C” variant (6 strains with 5 replicates each per sex) is shown in white. Underlying data can be found in S1 Data. Error bars represent the standard error of the mean. (A,B) Significance was assessed via a *t* test. (C,D) Significance was assessed using an ANOVA with sex, isofemale line, and population or variant at position 67 as factors. *P < 0.05, **P < 0.01, ***P < 0.005.

RNAi-knockdown and *CG9509*^*del*^ larvae were measured for larval growth rate and developmental timing (as measured by duration of the larval stage and wandering stage). *CG9509* expression had no effect on developmental timing (*t* test, *P* > 0.42, S4 Fig). In contrast, disruption of *CG9509* expression was associated with increased larval growth rate (Fig 4), and *CG9509* knockdown and *CG9509*^*del*^ larvae were significantly larger than control larvae at all stages (Figs 4 and 5F). We further examined adult body size, as measured by body weight and wing size (length and area), in RNAi*-*knockdown and *CG9509*^*del*^ flies. Disrupting *CG9509* expression increased body weight by 8%–15% (Fig 5A and 5B). Similarly, disruption of *CG9509* expression increased wing size by 4%–11% (Fig 5D and 5E), which was significant for all comparisons, except male wing area in *CG9509*^*del*^ flies. Next, we examined wing loading, which is the relationship between mass and wing area [39] and is dependent upon relative body size. To this end, we measured the wing load index (wet weight/right wing area) in *CG9509*^*del*^ and RNAi-knockdown flies. Disruption of *CG9509* expression increased wing loading in both males and females by 10%–35% (Fig 6A and 6B). The fact that similar results were obtained independently for the *CG9509*^*del*^ and RNAi-knockdown flies, which have different genetic backgrounds, suggests that the effect is a direct cause of losing *CG9509* expression and not an artifact of off-target gene silencing or mutations at other loci.

#### Adult body size and wing loading in a sub-Saharan and a cosmopolitan population

If selection favored a more active *CG9509* enhancer in cosmopolitan populations because of its negative effect on adult body size and/or wing loading, we would expect to find reduced adult body size and/or wing loading in cosmopolitan populations relative to sub-Saharan populations. To test this, we examined a sub-Saharan (Zambia) and a cosmopolitan (the Netherlands) population to determine if *CG9509* expression and enhancer variation could be associated with between-population differences in adult body size and/or wing loading. Contrary to the expectation, wing area and length were approximately 2%–10% reduced in the Zambian population in comparison to the Dutch population; however, these differences were not significant (ANOVA, *P* > 0.08 for both, S5 Fig). For adult body weight, there was a significant difference that also ran contrary to the expectation, with a significant reduction in the sub-Saharan population in comparison to the Dutch population (*P* < 10^−5^, S5 Fig). Wing loading, on the other hand, was significantly lower (approximately 3%–7.5%, *P* = 0.007, Fig 6C) in the Dutch population, which was in line with the expectation. However, it should be noted that these populations differ at more than just the *CG9509* locus; thus, at least some of the phenotypic differences observed between these populations could be due to correlated changes in *trans*-acting or other *cis*-acting factors.

#### Effect of *CG9509* expression on active ecdysone levels and the expression of 2 known growth regulators

**Fig 7:**
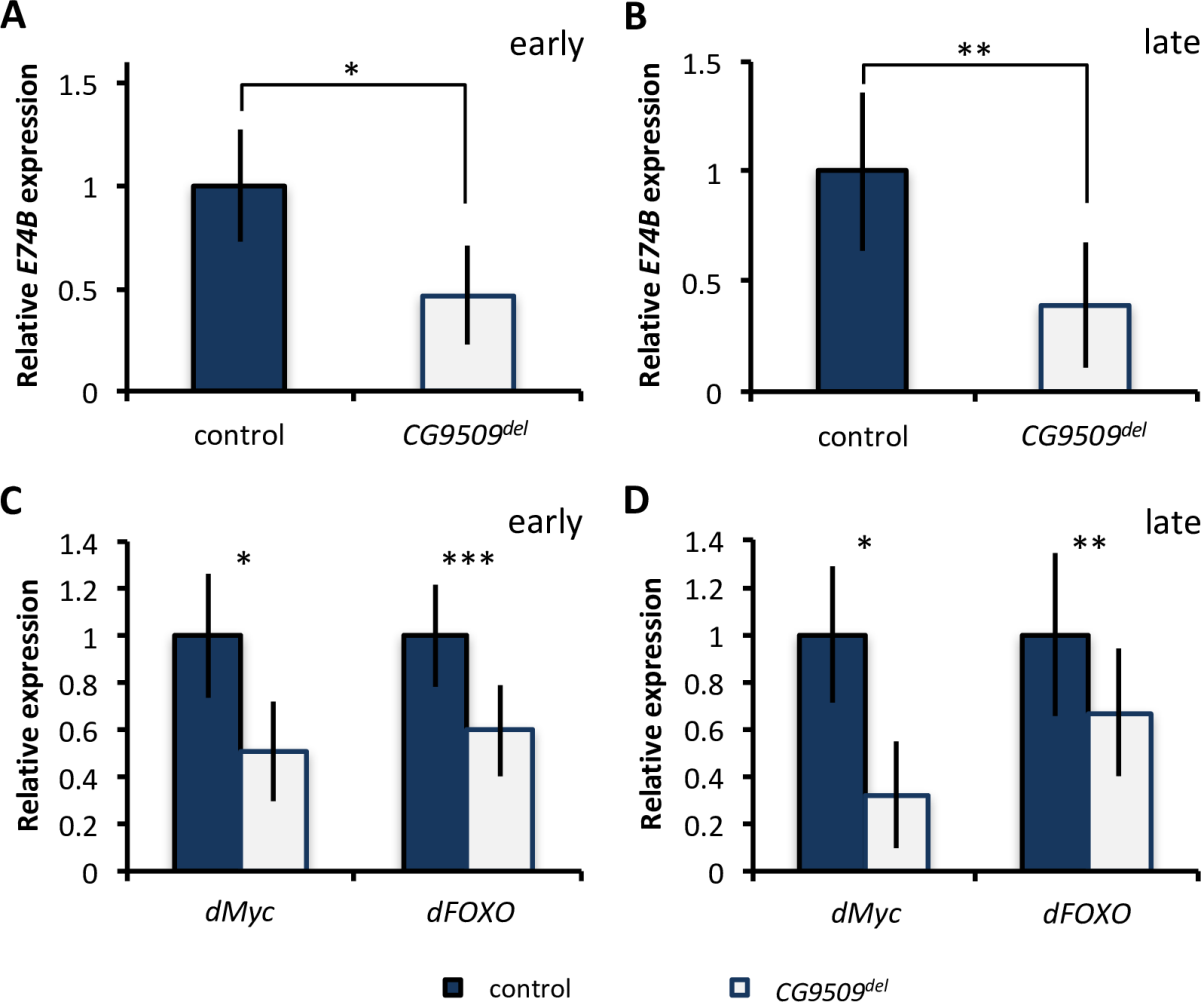
Effect of *CG9509* expression on active ecdysone levels and *dMyc* and *dFOXO* expression. (A, B) Levels of active ecdysone, approximated by relative *E74B* expression as measured by quantitative reverse transcription PCR (qRT-PCR) in (A) early and (B) late wandering third instar larvae in control (blue; *N* = 8 per stage) and *CG9509*^*del*^ (white; *N* = 4 per stage) flies. (C, D) Relative gene expression of *dMyc* and *dFOXO* in control (blue; *N* = 8 per stage) and *CG9509*^*del*^ (white; *N* = 4 per stage) (C) early wandering third instar larvae and (D) late wandering third instar larvae. Expression is shown relative to the control for each stage. Underlying data can be found in S1 Data and S2 Data. Error bars represent the standard error of the mean. Significance was assessed via a *t* test. *P < 0.05, **P < 0.01, ***P < 0.005.

*CG9509* has been predicted to be involved in metabolism of the steroid hormone ecdysone [30], pulses of which act as temporal signals during *D*. *melanogaster* development [40,41]. To estimate active ecdysone levels in early and late wandering third instar larvae, we measured expression of *E74B*, which is directly activated by ecdysone and often used as a readout of active ecdysone levels [42,43], in RNAi-knockdown and *CG9509*^*del*^ larvae. It must be noted, however, that because *E74B* expression is an indirect measure of active ecdysone levels, it may not correspond perfectly with actual ecdysone titers. When *CG9509* expression was disrupted, *E74B* expression decreased by approximately 50% in both early and late wandering third instar larvae (Fig 7A and 7B, S6 Fig). During larval development, ecdysone and insulin signaling interact to influence growth [44,45]. Integral to this interaction are the growth regulators forkhead box, sub-group O (dFOXO), and Myc (dMyc). We examined *dMyc* and *dFOXO* expression in early and late wandering third instar RNAi-knockdown and *CG9509*^*del*^ larvae, as these genes are known to have developmental stage-specific effects [40,44]. Disrupting *CG9509* expression resulted in a 40%–50% decrease in both *dMyc* and *dFOXO* expression in late wandering third instar larvae (Fig 7D and S6 Fig). Disrupting *CG9509* expression also decreased *dMyc* and *dFOXO* expression in early wandering third instar larvae; however, we could only detect this decrease in *CG9509*^*del*^ larvae (Fig 7C and S6 Fig). This discrepancy could be due to the decreased efficiency of *CG9509* RNAi-knockdown in this stage (76% versus >97% in all other examined stages). Thus, the association of *CG9509* expression with expression of these 2 important growth regulators occurs in at least 1 larval stage.

### Association of *CG9509* enhancer variants with body size and wing-loading variation

To determine if there was an association between SNP variation in the *CG9509* enhancer and variation in either body size or wing loading, we performed further analyses in the Dutch population, as well as in the reconstituted homozygotes, hemizygotes, and/or heterozygotes of the F2 generation from reciprocal crosses of individuals from either the Dutch or a Rwandan population (S1 Text). The genetic background of these reconstituted F2 individuals represents a mixture of the 2 original parental genomes, which allows us to better disentangle the effects of SNP variation within the *CG9509* enhancer from the effects of other variants in the genome (S1 Text), including *trans*-acting variants. Thus, association of *CG9509* enhancer SNP variants with body size or wing-loading variation within this shared background should represent true associations, rather than spurious associations caused by linked variation elsewhere in the genome.

#### Effect of the SNP at position 67 on body size and wing loading

To determine if body size and/or wing-loading variation was associated with the SNP at position 67, we examined adult body weight, wing size, and wing loading in the cosmopolitan population from the Netherlands. The high-expression "G" variant at this position is at intermediate frequency in this population (Fig 2B). Wing loading, body weight, and wing size (as measured by length and area) in flies with the high *CG9509* expression “G” variant were reduced by approximately 3%–10.5% in comparison to those with the ancestral “C” variant (ANOVA, *P* < 0.025 in all cases, Fig 6D and S5 Fig). These differences are driven by a shift in the phenotype distribution of “C” versus “G” variants rather than by a single isofemale line or measurement (S7 Fig). To further characterize the magnitude of the effect of carrying a “G” versus a “C” at position 67 on these phenotypes, we calculated partial eta squared (*η*^*2*^_*p*_), which is a standardized measure of effect size in which effect sizes on the magnitude of approximately 0.01, 0.06, and 0.14 are generally considered to represent small, medium, and large effect sizes, respectively [38]. In this cosmopolitan population, the variant at position 67 has a moderate (wing loading *η*^*2*^_*p*_ = 0.046) to large (wing area *η*^*2*^_*p*_ = 0.311, wing length *η*^*2*^_*p*_ = 0.487) effect on these traits. To exclude the possibility that these associations are caused by variation at a linked site, we calculated the degree of linkage disequilibrium, *r*^*2*^ [46], between position 67 and all SNPs, excluding singletons, within a 50-kb region centered around position 67 in this population. We did not detect any significant association between the variant at position 67 and any other SNP in this region (Fisher’s exact test, *P* > 0.06 for all comparisons, S5 Table). However, it should be noted that our analysis does not take into account potential variation in *trans*-acting factors that may also contribute to body size and wing-loading variation in this population.

To further confirm the association of body size and wing-loading variation with sequence variation at position 67, we measured body weight, wing size (length and area), and wing load index in the F2 offspring of a set of reciprocal crosses between a “G” variant and a “C” variant isofemale line from the Dutch population. Briefly, we performed reciprocal crosses between the “C” and “G” variant isofemale lines and then crossed the F1 offspring within each cross. In the F2 generation, we measured the phenotype and genotyped flies using a PCR- and restriction enzyme-based assay. Presence of the high-expression “G” variant was associated with reduced body weight, wing area, wing length, and wing loading, with the magnitude of reduction generally increasing with the number of “G” variant alleles (Table 2). This reduction was significant for body weight, wing length, and wing area (ANOVA, *P* < 0.015 for each comparison) and represents a medium-sized effect of the SNP variant at position 67 (body weight *η*^*2*^_*p*_ = 0.063, wing length *η*^*2*^_*p*_ = 0.102, wing area *η*^*2*^_*p*_ = 0.070) on these 3 traits in a cosmopolitan background (Table 2). On the other hand, this reduction was nonsignificant for wing loading (*P* = 0.317), corresponding to only a small-sized (*η*^*2*^_*p*_ = 0.011) effect of the SNP variant at position 67 on this trait in a cosmopolitan background (Table 2).

**Table 2:** Effect of single nucleotide polymorphism (SNP) variation at position 67 on adult body size and wing loading in F2 offspring of reciprocal crosses between a “G” variant and a “C” variant isofemale line from a Dutch population.

SNP 67^a^	Sex^b^	Weight (mg) ± SEM	Wing area (mm^2^) ± SEM	Wing length (mm) ± SEM	Wing load index (mg/mm^2^) ± SEM
G/G	F	1.534 ± 0.036	1.277 ± 0.020	2.129 ± 0.020	1.184 ± 0.025
G/C	F	1.560 ± 0.034	1.288 ± 0.014	2.157 ± 0.014	1.214 ± 0.027
C/C	F	1.565 ± 0.035	1.280 ± 0.016	2.153 ± 0.011	1.224 ± 0.024
G	M	1.204 ± 0.026	0.977 ± 0.013	1.833 ± 0.012	1.245 ± 0.026
C	M	1.324 ± 0.034	1.046 ± 0.019	1.904 ± 0.018	1.269 ± 0.026
	***P* value**^**c**^	0.012	0.011	0.002	0.317
	**η**^**2**^_**p**_^**d**^	0.063	0.07	0.102	0.011

^a^Nucleotide at position 67 in the *CG9509* enhancer. Males are hemizygous and therefore carry only 1 variant.

^b^M, male; F, female

^**c**^Significance was assessed using an ANOVA with sex, cross, and variant at the position of interest as factors. The effect of the SNP variant was assumed to be additive, with cosmopolitan homo- and hemizygotes assigned a value of 2, sub-Saharan homo- and hemizygotes assigned a value of 0, and heterozygotes assigned a value of 1.

^d^Effect size of the SNP at position 67 on the surveyed trait

#### Effect of the SNPs at positions 1063 and 1174 on body size and wing loading

The cosmopolitan variants at positions 1063 and 1174 are fixed in the surveyed cosmopolitan populations and absent or at too low of a frequency in the surveyed sub-Saharan populations (Fig 2B) to be able to detect an association between these SNPs and body size or wing-loading variation within 1 of the surveyed populations. For this reason, we utilized 3 isofemale lines isolated from a Rwandan population, where the cosmopolitan SNPs are at moderate frequency (9.5%–16%) [47]. Due to the low frequency and linkage of these SNPs in the Rwandan population, the effects of these positions were surveyed together using 3 isofemale lines that differed along a block spanning positions 1174, 1155, 1063, and 821–817 but were otherwise identical at the other positions of interest within the *CG9509* enhancer (Fig 2B, S1 Text). While we are unable to separate the effects of the SNP variants at these 4 positions, we have shown that the variants at positions 1155 and 821–817 do not contribute to *CG9509* expression divergence (Fig 3).

Two sets of reciprocal crosses between 2 isofemale lines with a cosmopolitan “A” and “T” at positions 1174 and 1063, respectively, (hereafter designated “AT” variant) and an isofemale line with a sub-Saharan “C” and “G” at these positions (hereafter designated “CG” variant” (S1 Text) were performed, and body weight, wing size (length and area), and wing load index were measured in the F2 offspring. Briefly, we performed separate reciprocal crosses between the 2 “AT” variant isofemale lines and the “CG” variant isofemale line and then crossed the F1 offspring within each cross. Next, in the F2 generation, we measured the phenotype in homo- and hemizygotes and genotyped flies using sequencing. Presence of the cosmopolitan “AT” variant was associated with reduced body weight, wing area, wing length, and wing loading (Table 3). This reduction was significant for body weight and wing area (ANOVA, *P* < 0.007 for both comparisons) and moderately significant for wing length (ANOVA, *P* = 0.052), corresponding to a moderate (wing length *η*^*2*^_*p*_ = 0.0315) or medium-sized (body weight *η*^*2*^_*p*_ = 0.069, wing area *η*^*2*^_*p*_ = 0.061) effect of the variants at positions 1174 and 1063 on these 3 traits in this population (Table 3). On the other hand, this reduction was not significant for wing loading (*P* = 0.128), corresponding to only a small-sized (*η*^*2*^_*p*_ = 0.020) effect of variants at positions 1174 and 1063 on this trait in this population (Table 3).

**Table 3:** Effect of single nucleotide polymorphism (SNP) variation at positions 1174 and 1063 on adult body size and wing loading in F2 offspring of reciprocal crosses between 2 cosmopolitan “AT” variant isofemale lines and a sub-Saharan “CG” variant line from a Rwandan population.

SNPs 1174 and 1063^a^	Sex^b^	Weight (mg) ± SEM	Wing area (mm^2^) ± SEM	Wing length (mm) ± SEM	Wing load index (mg/mm^2^) ± SEM
AT/AT	F	1.549 ± 0.028	1.360 ± 0.015	2.219 ± 0.015	1.142 ± 0.024
CG/CG	F	1.617 ± 0.033	1.394 ± 0.014	2.256 ± 0.014	1.163 ± 0.023
AT	M	1.154 ± 0.027	1.016 ± 0.013	1.905 ± 0.013	1.140 ± 0.029
CG	M	1.234 ± 0.020	1.066 ± 0.008	1.931 ± 0.009	1.164 ± 0.022
	***P* value**^**c**^	0.003	0.0071	0.052	0.128
	**η**^**2**^_**p**_^**d**^	0.069	0.061	0.032	0.012

^a^Nucleotide at positions 1174 and 1063, respectively, in the *CG9509* enhancer. Males are hemizygous and therefore carry only 1 variant.

^b^M, male; F, female

^**c**^Significance was assessed using an ANOVA with sex, cross, and variant at the position of interest as factors. The effect of the SNP variant was assumed to be additive, with cosmopolitan homo- and hemizygotes assigned a value of 2, sub-Saharan homo- and hemizygotes assigned a value of 0, and heterozygotes assigned a value of 1.

^d^Effect size of positions 1174 and 1063 on the surveyed trait

## Discussion

We have shown that the between-population expression divergence of *CG9509* occurs in both larvae and adults (Fig 1) and is driven by nucleotide polymorphism within the *CG9509* enhancer (Fig 3). We identified 3 SNPs that can account for the majority of the expression divergence, 2 of which (at positions 1174 and 1063, Fig 2B) only affect expression in larvae and have a relatively small effect on larval expression (Fig 3C). The third SNP (position 67, Fig 2B) accounts for the majority of the expression divergence in both adults and larvae (Fig 3). We propose that selection on the *CG9509* enhancer occurred in 2 phases. First, the derived variants at positions 1174 and 1063 were the targets of the previously identified selective sweep [12]. These variants are fixed in cosmopolitan populations but absent or at low frequency in ancestral, sub-Saharan African populations (Fig 2B). Thus, this sweep likely occurred during or shortly after *D*. *melanogaster*’s expansion out of Africa but before the separation of European and Asian populations. A single haplotype spans positions 1174 and 1063 in cosmopolitan populations, suggesting that both derived variants were fixed by a single selective sweep. The derived variants at these positions are also present at low frequency in central African populations [47], suggesting that selection acted on standing variation. In a second step, we propose that the large-effect derived variant at position 67 arose as a new mutation on the selected haplotype and rose to intermediate frequency more recently in cosmopolitan populations. Consistent with this view, the derived variant is absent from sub-Saharan and central African populations (Fig 2B) [47]. It has been proposed that advantageous regulatory mutations with large effects are likely to display overdominance and, thus, remain polymorphic within populations [48]. The large effect of the derived variant at position 67 on *CG9509* expression (Fig 3 and S1 Fig) and its intermediate frequency in cosmopolitan populations (Fig 2B) are consistent with this model. However, other causes for its maintenance at intermediate frequency, such as sexual antagonism, temporally varying selection, or the interaction of alleles at multiple loci, are also possible [48–51].

Until now, *CG9509*’s function and, therefore, the organismal phenotype(s) affected by variation in its expression have remained unknown. Here, we used RNAi-mediated knockdown of *CG9509* expression and a newly identified *CG9509* hypomorph allele to show that increased *CG9509* expression is associated with reduced wing loading (Fig 6). Wing loading in a Dutch population was associated with polymorphism at position 67 (Fig 6D), and we also found that flies from Zambia had greater wing loading than those from the Netherlands (Fig 6C). When we surveyed wing loading in the F2 offspring of crosses between fly strains containing either cosmopolitan or sub-Saharan *CG9509* enhancer variants affecting expression, wing loading varied in the expected direction (Tables 2 and 3), but this variation was small (approximately 1%–3%) and nonsignificant (Tables 2 and 3). Some of this discrepancy between our findings in Dutch and Zambian populations versus F2 offspring may be due to *trans*-acting variation present among the inbred lines from different populations. This variation should be greatly reduced in the F2 offspring, which share a more homogenous *trans* environment after a generation of recombination. However, we did detect significant associations between these variants and both body weight and wing size (Tables 2 and 3). Our inability to detect significant associations with wing loading may be a result of this trait being a ratio of 2 measurements, which increases trait variance and reduces statistical power. It is also possible that the effect size is too small to be detected as significant in our experimental design. Previous studies have shown that even small changes in flight load can lead to differences in flight performance [52–53], especially at low temperatures [53], and therefore impact fitness.

Consistent with our findings, several studies have documented clinal variation in wing loading among *Drosophila* populations across multiple continents [39,53–56], with reduced wing loading at higher latitudes, and this cline is thought to be maintained by selection. In *Drosophila*, and indeed all flying animals, relative size is important for flight aerodynamics. Furthermore, previous microarray comparisons of gene expression have found overexpression of muscle-related genes (including flight muscle components) in flies from Zimbabwe relative to those from the Netherlands [24,25], suggesting that flies in the ancestral range require a greater investment in flight muscle. This suggests that improved flight ability may have been an important adaptation as *D*. *melanogaster* expanded its species range, and selection on the *CG9509* enhancer likely favored the reduction in wing loading conferred by increased larval *CG9509* expression as *D*. *melanogaster* expanded out of Africa. *Drosophila* wing beat frequency and power output decrease as temperature decreases, resulting in reduced flight ability at cooler temperatures [53], and previous studies suggest that reduced wing loading may help counteract this effect [39,57–58]. Thus, improved flight ability may represent an adaptation to lower temperatures in the derived species range; however, general improvement of flight ability could also be adaptive. The energy conserved by improved flight ability could be used for other processes or aid in survival when resources are scarce. Improved flight ability might also aid in predator evasion or dispersion, which may have helped facilitate *D*. *melanogaster*’s expansion to new territories. However, it is important to note that, although selection for reduced wing loading represents a plausible scenario for adaptive regulatory evolution at the *CG9509* locus, we cannot rule out the possibility that selection acted on an unobserved, pleiotropic trait associated with variation in the *CG9509* enhancer.

We have shown that increased *CG9509* expression is associated with reduced larval growth (Fig 4) and adult body size (Fig 5) and were able to associate body size variation with *CG9509* enhancer sequence variation (Tables 2 and 3), with the high-expression, cosmopolitan variants associated with decreased body size. We also showed that weight is reduced in a sub-Saharan population in comparison to a Dutch population (S5 Fig). However, these results are contrary to expectations if selection acted on the *CG9509* enhancer to reduce body size in cosmopolitan populations but in line with well-documented, latitudinal body size clines that are thought to be maintained by selection [55,59]. We additionally showed that increased *CG9509* expression is associated with increased levels of the maturation hormone ecdysone (Fig 7A and 7B, S6 Fig). Most likely, the increased active ecdysone levels result in the reduced larval growth rate and a subsequently smaller body size, since the antagonistic interaction of ecdysone with insulin signaling is known to suppress larval growth [43,60], which in turn reduces adult body size. However, the mechanism through which *CG9509* expression adjusts larval growth to reduce wing loading remains unknown. The effect on wing loading (Fig 6) is at least in part due to *CG9509*’s effect on active ecdysone levels (Fig 7A and 7B, S6 Fig), as ecdysone plays a key role in regulating proportional growth and coordinating the growth of individual organs with each other as well as with the entire body [61].

*CG9509* is expressed in the larval fat body [62], which acts as a coordinator of larval growth [40,41,44]. Ecdysone signaling specifically in the fat body antagonizes insulin signaling in part via down-regulation of the positive growth regulator dMyc and translocation of the negative growth regulator dFOXO to the nucleus, where it activates the expression of target genes [44,45,63]. When we knocked down *CG9509* expression, we found a decrease in both *dMyc* and *dFOXO* expression in late wandering third instar larvae (Fig 7D and S6 Fig), which is the stage during which the peak of the final and largest larval ecdysone titer occurs, signaling the onset of pupariation [40,41]. We detected a similar decrease in the early wandering third instar larval stage, which coincides with another, smaller ecdysone peak [40,41], but only in *CG9509*^*del*^ larvae (Fig 7D). The reduction in *dFOXO* transcript expression is interesting, as it is dFOXO protein localization that suppresses growth [44,45,63]. However, a study documenting insulin/TOR network transcriptional variation found strong covariance for *dFOXO* transcript abundance and the expression of dFOXO-affected genes [64]. Thus, the expression of genes downstream of dFOXO may also be affected. The reduction in *dMyc* expression (Fig 7D) is counterintuitive, since its up-regulation in the fat body is expected during *CG9509* expression knockdown. However, the effect of ecdysone on *dMyc* expression is both stage- and tissue-specific [40]; thus, the expression decrease is likely in another tissue and may represent a part of the mechanism through which *CG9509* expression adjusts proportional growth to affect wing loading. Interestingly, previous studies have documented negative correlations of the expression of growth-associated genes with stress tolerance [65,66], which we also found for *CG9509* expression (Table 1). However, this correlation could simply be a by-product of body size, which has been shown to correlate with stress tolerance in *Drosophila* [67].

We identified 3 SNPs that account for the majority of *CG9509* expression divergence observed between cosmopolitan and sub-Saharan *D*. *melanogaster* (Figs 1 and 3). Indeed, when we mutated these SNPs, we were able to recover 100% of this expression divergence in the cosmopolitan background (Fig 3), although we also found evidence that unidentified SNPs in the *CG9509* enhancer have epistatic effects on expression in the sub-Saharan background in larvae and the cosmopolitan background in adults (Fig 3). However, these epistatic effects are small relative to the magnitude of expression divergence that could be attributed to the 3 SNPs of major effect. Furthermore, the context-dependent nature of these effects makes it unlikely that they have been targets of positive selection, which acts most efficiently on additive genetic variation [68]. While we assume that the identified SNPs exert their effects on gene expression through interactions with *trans*-acting factors, the specific *trans*-acting factors that are involved remain unknown. To identify potential transcription factors that might interact with the identified SNPs, we scanned representative cosmopolitan and sub-Saharan *CG9509* enhancer sequences for predicted transcription factor binding sites (TFBSs) [69]. All of the identified SNPs overlapped with at least 1 predicted TFBS, and for each SNP, differential binding (absence or a lower binding score in 1 sequence) was predicted for 2–8 TFBS matrix models (S2 Table). The majority of the identified transcription factors are known to be involved in developmental regulation and morphogenesis, including several forkhead box factors, the Iroquois complex genes, hairy, Distal-less, slow border cells, and twist [70–74]. Several, such as fork head and the Broad-Complex [75,76], are also known to be involved in insulin and/or ecdysone signaling.

It is important to elucidate both the mechanisms behind and the selective forces driving the adaptive divergence of *cis*-regulatory elements, as these examples help us to understand the genetic basis of phenotypic evolution, which can give further insights about biodiversity. Our results provide evidence that in cosmopolitan populations of *D*. *melanogaster*, positive selection has acted on 3 SNPs within the *CG9509* enhancer to increase *CG9509* expression and thereby reduce wing loading. While 2 of these SNPs appear to be the targets of a completed selective sweep, the third, which has the largest effect on *CG9509* expression, has been maintained at intermediate frequency, suggesting that it has been subject to another mode of selection. Using natural variation, a mutant allele, and RNAi, we provide the first experimental evidence of *CG9509*’s function. We show that its expression influences larval and adult body size, as well as the ratio of wing-to-body size. We propose that the reduced wing loading conferred by elevated *CG9509* expression represents an adaptation to improve flight ability as *D*. *melanogaster* expanded out of Africa. Because of the remarkable body size increase seen in *CG9509*^*del*^ larvae and adults, we propose that the gene be named *fezzik* (*fiz*) after the giant character in *The Princess Bride*.

## Materials and methods

### *D*. *melanogaster* lines

All flies were maintained as inbred, isofemale lines under standard conditions (22°C, 14 hours light:10 hours dark cycle, cornmeal-molasses medium). The *phiX-86Fb* stock [77], containing a mapped *attP* site on the third chromosome (cytological position: 3R 86F), was obtained from the Bloomington Stock Center (Indiana, United States) and used for phiC31 site-specific integration.

#### Population samples

Expression of *CG9509* was surveyed in isofemale lines derived from the following locations: Leiden, the Netherlands (12 lines); Kuala Lumpur, Malaysia (11 lines); Cairo, Egypt (12 lines); Siavonga, Zambia (11 lines); and Lake Kariba, Zimbabwe (11 lines). Body size and wing-loading assays were performed in the Dutch and Zambian populations. To survey the effect of *CG9509* SNP variants on body size and wing-loading variation, a series of reciprocal crosses and subsequent body size and wing-loading assays were performed using 2 Dutch lines and 3 lines (RG11N, RG25, and RG28) from a population in Gikongoro, Rwanda [47]. All of the surveyed populations, with the exception of Rwanda, were used in a previous study of adult expression and sequence variation associated with the *CG9509* enhancer region [31]. The Zimbabwean and Dutch populations were also used in a previous study of sequence and expression variation associated with the *CG9509* enhancer region [12] as well as genome-wide expression studies [24–26,28].

#### Crosses to test the association between *CG9509* enhancer SNPs and phenotype

Body size and wing-loading assays were performed on the F2 offspring of 3 pairs of reciprocal crosses. Reciprocal crosses of 30–40 females and 15–20 males were performed for each pair. Forty to fifty F1 progeny were allowed to randomly mate, and phenotypes were measured in the F2 generation. Adults for phenotype measurements were staged as described in S1 Text. Crosses were performed using 2 Dutch lines with either a “C” or a “G” variant at position 67 but identical at other sites of interest in the *CG9509* enhancer (Fig 2B) and 3 Rwandan lines (RG11N, RG25, and RG38) [47] containing either cosmopolitan or sub-Saharan variants at positions 1174, 1155, 1063, and 821–817 (Fig 2B, S1 Text). Flies were genotyped using either a PCR followed by restriction enzyme digestion for the Dutch crosses or sequencing for the Rwandan crosses (S1 Text).

#### *CG9509* hypomorph and knockdown lines

*CG9509*, *E74B*, *dFOXO*, and *dMyc* expression analyses as well as body size, larval growth rate, developmental timing, wing-loading, and tolerance assays were performed on flies in which the open reading frame (ORF) of the *CG9509* gene was disrupted and/or in which *CG9509* expression was knocked down by RNAi. We discovered a mutant *CG9509* allele as a naturally occurring variant in an isofemale line from a Munich population. In this line (*CG9509*^*del*^), a deletion introduces a frameshift that leads to a premature stop codon 232 amino acids into the *CG9509* ORF (S2 Fig). Our quantitative reverse transcription PCR (qRT-PCR) assay (see below) was able to detect the expression of *CG9509* mRNA in this line, but only at very low levels (S2 Fig), suggesting that it is degraded by the nonsense-mediated decay pathway. Given the disrupted ORF and the very low expression, we assume that *CG9509* function is greatly reduced in this line. For this reason, we refer to it as a hypomorph allele. As a control, 4 lines from the same Munich population, showing representative *CG9509* expression for the population, were used. We further confirmed that the observed phenotypes are likely driven specifically by the *CG9509* hypomorph allele rather than variation located elsewhere in the genome (S1 Text).

The knockdown of *CG9509* expression was achieved using an RNAi construct under the control of the yeast GAL4/UAS system. A *D*. *melanogaster* line producing a hairpin RNA complementary to *CG9509* mRNA under the control of a UAS (RNAi-*CG9509*, transformant ID: 107089) as well as a line containing an empty vector at the same genomic location (UAS^-^, transformant ID: 60100), which we used as a control, were obtained from the Vienna *Drosophila* Resource Center (Vienna, Austria) [78]. The RNAi-*CG9509* and UAS^-^ lines were crossed to an *Act5C-GAL4*/*Cyo* driver line, and the progeny were used in subsequent body size, larval growth rate, developmental timing, wing-loading, and tolerance assays, as well as in expression analyses. Using qRT-PCR, *CG9509* expression knockdown efficiency was estimated to be 98.6% for adult females, 98.9% for adult males, 76.0% for early wandering third instar larvae, and 97.6% for late wandering third instar larvae.

### Expression analysis

In adults, *CG9509* expression is highly enriched in the Malpighian tubule, while in larvae, it is enriched in the Malpighian tubule and fat body [62]; therefore, we surveyed expression in whole flies and larvae. Total RNA was extracted from 3–5 adult males (aged 4–6 days) or 1–3 early or late third instar wandering larvae, and a DNAse I digestion was performed using the MasterPure RNA Purification Kit (Epicentre; Madison, Wisconsin, US). Two biological replicates were performed for each line and/or stage. Using random hexamer primers and Superscript III reverse transcriptase (Invitrogen; Carlsbad, California, US), 3 μg total RNA for each replicate was reverse transcribed following the manufacturer’s protocol. TaqMan Gene Expression Assays (Invitrogen; Carlsbad, California, US) were then performed on the resulting cDNA using probes specific to *CG9509* (Dm01838873_g1), *dFOXO* (Dm02140207_g1), *dMyc* (Dm01843706_m1), and/or *E74B* (Dm01793592_m1) as well as a probe specific to the ribosomal protein gene *RpL32* (Dm02151827_g1), which was used as an endogenous control. The ΔΔCt method was used to calculate normalized gene expression [79]. Briefly, for each biological replicate, the average threshold cycle (Ct) of 2 technical replicates was measured, and ΔCt was calculated as the mean Ct difference between the probe of interest and the *RpL32* probe. The fold-change difference in expression relative to the Zimbabwe population for population comparisons or the control lines for *CG0509* hypomorph and knockdown comparisons was then calculated as 2^–(ΔCtX–ΔCtY)^, where ΔCtX is the mean ΔCt value for each biological replicate of the line of interest and ΔCtY is the mean ΔCt value of either the Zimbabwe or control lines. Significance was assessed with a *t* test. When more than 3 comparisons were made using the same data, a Bonferroni multiple test correction was applied.

### Transgenic reporter gene assays

The *CG9509* enhancer region, spanning coordinates 14,909,008–14,910,193 of the X chromosome (release 6), was PCR-amplified from 2 cosmopolitan strains and 1 sub-Saharan strain as described in [12] and cloned into the *pCR2*.*1-TOPO* vector (Invitrogen; Carlsbad, California, US). The effects of 6 sub-Saharan sequence variants (positions 67, 765, 821–817, 1063, 1155, and 1174; Fig 2B) in the cosmopolitan background were examined. The sub-Saharan African variants were introduced into the cosmopolitan sequence using either standard cloning techniques or site-directed mutagenesis (S1 Text) [80]. For sites shown to affect reporter gene expression, the cosmopolitan variants were introduced into the sub-Saharan enhancer, and constructs with all contributing sites were generated in both a cosmopolitan and sub-Saharan background using site-directed mutagenesis (S1 Text) for a total of 13 reporter gene constructs. The original and the mutated enhancer sequences were confirmed via sequencing (S1 Text). The *Escherichia coli LacZ* coding region was then inserted downstream of the *CG9509* enhancer sequence, and both were introduced into the *pattB* integration vector [77] using standard cloning techniques (S1 Text). The *pattB* vectors containing the *CG9509* enhancer and the *LacZ* reporter gene were microinjected into early-stage embryos of the *phiX-86Fb* (*attP* site at cytological band 86F) strain [77], which contains a stable source of *phiC31* integrase on the X chromosome. After microinjection, surviving flies were crossed to a *white*^-^ strain to remove the integrase source, and stable lines homozygous for each of the constructs were established. A subset of the microinjections was performed by Rainbow Transgenic Flies (Camarillo, CA, US).

In adults, *CG9509* expression is highly enriched in the Malpighian tubule, while in larvae, it is enriched in the Malpighian tubule and fat body [62], and adult reporter gene in whole flies has been shown to be a good proxy for Malpighian tubule expression [12]; therefore, we surveyed reporter gene expression in whole flies and larvae. For each reporter gene construct, β-galactosidase activity was measured in groups of 15 adult 4–6-day-old males or females or 8 late wandering third instar larvae. Soluble proteins were extracted, and a β-galactosidase activity assay was performed as described in [81] with the following modifications: flies or larvae were frozen with liquid nitrogen and homogenized before the addition of 200 μl of the 0.1 M Tris-HCl, 1 mM EDTA, and 7 mM 2-mercaptoethanol buffer (pH 7.5). β-galactosidase activity was measured spectrophotometrically by following the change in absorbance at 420 nm at 37°C. Four to eight biological replicates were performed per stage or sex. Significance was assessed using a *t* test, and a Bonferroni multiple test correction was applied for each stage and sex. To better understand the effect of position 67 on reporter gene expression in adults, an ANOVA using sex, background, the variant at position 67, and the interaction between the variant at position 67 and background, sex, and the other tested sites within the *CG9509* enhancer was performed.

### Body size assays

#### Weight

The wet weight of flies was measured in groups of 25 males or females or, for F2 offspring, single flies. Groups of flies were lightly anesthetized with CO_2_ and placed in preweighed 1.5 mL Eppendorf tubes on ice for 5 minutes before being weighed on a Mettler H51 scale (d = 0.01 mg, error = 0.05 mg). The weight of 25 flies was then calculated as the weight of 25 flies and tube minus the weight of the tube. For each line and sex, 4 replicates were performed for population comparisons, 5–7 replicates were performed for the *CG9509*^*del*^ and RNAi-knockdown lines, and 4–5 replicates were performed for all control lines. For F2 offspring, 13–35 individual flies were weighed for each genotype and sex. Significance was assessed using a *t* test for *CG9509*^*del*^ and RNAi comparisons. For population comparisons, significance was assessed using an ANOVA with sex, isofemale line, and population or variant at position 67 as factors. For F2 offspring comparisons, significance was assessed using an ANOVA with sex, cross, and SNP variant(s) as factors. The effect of the SNP variant(s) was assumed to be additive, with cosmopolitan homo- and hemizygotes assigned a value of 2, sub-Saharan homo- and hemizygotes assigned a value of 0, and heterozygotes assigned a value of 1.

#### Wing size

For each fly, the right wing (or the left wing if the right wing was damaged) was dissected in isopropanol, mounted in Euparal (Carl Roth; Karlsruhe, Germany), and allowed to dry at least 1 week before being photographed. Wings were photographed using a Nikon D5100 camera and compound microscope. Images were analyzed in ImageJ [82]. A piece of millimeter paper was included in all images for scale. Wing length was measured in a straight line from the humeral-costal break to the third longitudinal vein, and wing area was estimated as previously described [55]. For each line and sex, wing size was measured for 5 flies for population comparisons and 10–15 biological replicates per sex for RNAi-*CG9509*/*Act5C*-*GAL4*, UAS^-^/*Act5C*-*GAL4*, and *CG9509*^*del*^ and control lines. For F2 offspring, 11–35 wings were measured for each genotype and sex. Significance was assessed using a *t* test for *CG9509*^*del*^ and RNAi comparisons. For population comparisons, significance was assessed using an ANOVA with sex, isofemale line, and population or variant at position 67 as factors. For F2 offspring comparisons, significance was assessed using an ANOVA with sex, cross, and SNP variant(s) as factors. The effect of the SNP variant(s) was assumed to be additive, with cosmopolitan homo- and hemizygotes assigned a value of 2, sub-Saharan homo- and hemizygotes assigned a value of 0, and heterozygotes assigned a value of 1.

### Wing-loading assays

Wing load index was calculated as the wet weight of a fly divided by the area of its right wing. Flies were lightly anesthetized with CO_2_ and placed in preweighed 1.5 mL Eppendorf tubes on ice for 5 minutes before being weighed on a Mettler H51 scale (d = 0.01 mg, error = 0.05 mg). The weight of a fly was then calculated as the weight of the fly and tube minus the weight of the tube. For each fly, the right wing (or the left wing if the right wing was damaged) was then dissected, and the wing area was estimated as described above. For each line and sex, wing loading was measured for 5 flies for population comparisons and 10–15 flies for RNAi-*CG9509*/*Act5C*-*GAL4* and *CG9509*^*del*^ lines as well as their respective control lines. For F2 offspring, wing loading was measured for 11–35 flies for each genotype and sex. Significance was assessed using a *t* test for *CG9509*^*del*^ and RNAi comparisons. For population comparisons, significance was assessed using an ANOVA with sex, isofemale line, and population or variant at position 67 as factors. For F2 offspring comparisons, significance was assessed using an ANOVA with sex, cross, and SNP variant(s) as factors. The effect of the SNP variant(s) was assumed to be additive, with cosmopolitan homo- and hemizygotes assigned a value of 2, sub-Saharan homo- and hemizygotes assigned a value of 0, and heterozygotes assigned a value of 1.

### Larval growth assays

To assess larval growth rate, larval volume was measured in the following stages: second instar approximately 48 hours after egg laying (AEL), early third instar (72 hours AEL), early wandering third instar (110 hours AEL), and late wandering third instar (116 hours AEL). Larvae were staged as described in S1 Text. Before imaging, larvae were placed on ice for at least 5 minutes. Larvae were photographed using a Nikon D5100 camera and a compound microscope, and images were analyzed in ImageJ [82]. A piece of millimeter paper was included in all images for scale. Larval volume was calculated as 4/3π(L/2)^2^(d/2), where L = length and d = diameter [60]. For each stage and line, larval volume was measured in 15–20 larvae for RNAi-*CG9509* /*Act5C*-*GAL4* and *CG9509*^*del*^ lines as well as their respective controls. Significance at each larval stage was assessed using a *t* test, and a Bonferroni multiple test correction was applied.

### Developmental timing assays

As a measure of developmental timing, the time from the first instar larval stage to pupariation and the duration of the wandering stage were measured. As described in S1 Text, flies were allowed to lay eggs for 12 hours, and first instar larvae were collected. Larvae were transferred in groups of 50 to cornmeal-molasses medium and allowed to mature. In order to measure the duration of the larval stage (L1 to pupariation), pupariation was recorded every 2 hours for 25–110 larvae per line. In order to measure the duration of the wandering stage, larvae were screened for onset of wandering behavior every hour and transferred individually to a petri dish containing moistened filter paper. Pupariation was recorded every hour for 10–50 larvae per line. Both assays were performed at 25°C to prevent fluctuations in developmental timing due to temperature.

### Tolerance assays

DDT, malathion, ethanol, and cold tolerance assays were performed using RNAi-*CG9509*/*Act5C-*GAL4 and UAS^-^/Act5C-GAL4 flies. For DDT, malathion, and ethanol tolerance assays, for each line, sex, and concentration, 6–8 tolerance chambers with 20 flies each were exposed to 4 concentrations of a compound, and mortality was measured as the number of flies dead or unable to move after 30 minutes (malathion), 2 hours (DDT), or 48 hours (ethanol). For ethanol tolerance assays, tolerance chambers consisted of a plastic vial (diameter = 25 mm, height = 95 mm) with compressed cotton at the bottom containing 2.5 ml ethanol solution supplemented with 5% sucrose and sealed with a cork. For DDT and malathion assays, tolerance chambers consisted of glass vials (h = 5 cm, r = 1.65 cm) in which 200 μl of DDT (Dr. Ehrenstorfer; Augsburg, Germany) or malathion (Dr. Ehrenstorfer; Augsburg, Germany) diluted in acetone was swirled until the acetone dried; the vials were allowed to dry an additional hour before addition of flies and were sealed with compressed cotton soaked in 5% sucrose solution. For all assays, 2–3 control chambers containing only 5% sucrose solution were also tested. The data for each assay were fit to a generalized linear model using concentration, line, and sex as factors (unless sex was not significant, in which case it was removed from the model) and a quasibinomial distribution using the glm function in R [83]. For cold tolerance assays, for each line and sex, 25 groups of 5 flies were exposed to an ice water bath for 5 hours, and the time in minutes until each fly had recovered from chill coma (able to stand upright again) was recorded. The mean recovery time for each vial was calculated, and a *t* test was applied to assess significance.

## Supporting information

S1 Data Data underlying manuscript figures. (XLSX)

S2 Data Data underlying supporting information figures. (XLSX)

S1 Fig Larval *CG9509* expression. Relative expression in late wandering third instar larvae in (A) the Netherlands (Net.), Malaysia (Mal.), Egypt (Egy.), Zimbabwe (Zim.), and Zambia (Zam.) populations (*N* = 10–12 isofemale strains per population with 2 biological replicates per strain). Blue bars represent cosmopolitan populations, and white bars represent sub-Saharan populations. (B) Relative expression in each population represented according to the variant at position 67. The high-expression, cosmopolitan “G” variant is shown in blue, and the low*-*expression, sub-Saharan “C” variant is shown in white. For simplicity, Zambian and Zimbabwean expression are presented together as sub-Saharan African (Afr.) expression. Underlying data can be found in S1 Data. Error bars indicate the standard error of the mean. Differences between populations were tested by a *t* test, and a Bonferroni multiple test correction was applied. ns, not significant; ●*P* < 0.10, *P < 0.05, **P < 0.01, ***P < 0.005.
(PDF)

S2 Fig *CG9509*^*del*^ line details. (A) Relative expression of *CG9509* in the *CG9509*^*del*^ line as determined by quantitative reverse transcription PCR (qRT-PCR). For comparison, the average expression of the population in which the *CG9509*^*del*^ line was discovered (Munich) is shown. Underlying data can be found in S1 Data. Error bars represent the standard error of the mean. (B) DNA sequence alignment spanning the deletion within the *CG9509*^*del*^ coding region. Sequences of flies from the *CG9509*^*del*^ source population in Munich (MU) are shown for comparison. (C) Amino acid alignment spanning the frameshift within the *CG9509*^*del*^ coding region. Sequences of flies from the *CG9509*^*del*^ source population are shown for comparison (MU). The asterisk indicates a stop codon.
(PDF)

S3 Fig Effect of *CG9509* expression on insecticide, ethanol, and cold tolerance. Adult (A) DDT, (B) malathion, (C) ethanol (*N* = 6–8 replicates per line, sex, and concentration), and (D) cold tolerance assay results (*N* = 25 per line and sex) for control (blue lines or bars) and RNAi-*CG9509* (gray hatched lines or white bars) flies. Underlying data can be found in S2 Data. In panels A–C, significance was assessed using a generalized linear model with a quasibinomial distribution. In panel D, significance was assessed using a *t* test. ns, not significant; *P < 0.05, **P < 0.01, ***P < 0.005.
(PDF)

S4 Fig Effect of *CG9509* expression on developmental timing. (A, B) Duration of larval stage in (A) control (blue; *N* = 25) and RNAi-*CG9509* (white; *N* = 51) flies and (B) control (blue; *N* = 242) and *CG9509*^*del*^ (white; *N* = 107) flies. (C, D) Duration of wandering stage in (C) control (blue; *N* = 16) and RNAi-*CG9509* (white; *N* = 40) flies and (D) *CG9509*^*del*^ (white; *N* = 10) and control (blue; *N* = 40) flies. Underlying data can be found in S2 Data. Error bars represent the standard deviation. The knockdown and hypomorph lines were not significantly different from their respective control lines for either stage (*t* test; *P* > 0.4 for all comparisons).
(PDF)

S5 Fig Adult body size in natural populations. (A) Body weight per 25 flies (*N* = 10–12 isofemale lines per population with 4 replicates per sex), (C) wing length (*N* = 10–12 isofemale lines per population with 4 replicates per sex), and (E) wing area in a Dutch (blue bars) and a Zambian (white bars) population (*N* = 10–12 isofemale lines per population with 4 replicates per sex). (B) Body weight per 25 flies (*N* = 6 isofemale lines per variant with 4 replicates per sex), (D) wing length (*N* = 6 isofemale lines per variant with 4 replicates per sex), and (F) wing area in a Dutch population separated according to the variant at position 67 (*N* = 6 isofemale lines per variant with 4 replicates per sex). The derived, high-expression “G” is shown in blue, and the ancestral, low-expression “C” variant is shown in white. Error bars indicate the standard error of the mean. Underlying data can be found in S2 Data. Significance was assessed with an ANOVA using sex, isofemale line, and population or the variant at position 67 as factors (shown in black). Significance was additionally assessed in both populations simultaneously with population and the variant at position 67 included as factors (shown in red). ns, not significant; ●0.05 < *P* < 0.10, *P < 0.05, **P < 0.01, ***P < 0.005.
(PDF)

S6 Fig Effect of *CG9509* expression on active ecdysone levels and *dMyc* and *dFOXO* expression. (A, B) Levels of active ecdysone, approximated by relative *E74B* expression as measured by quantitative reverse transcription PCR (qRT-PCR) in (A) early and (B) late wandering third instar larvae in control (blue) and RNAi-*CG9509* (white) flies (*N* = 9–10 per line). (C, D) Relative gene expression of *dMyc* and *dFOXO* in control (blue) and RNAi-*CG9509* (white) (C) early wandering third instar larvae and (D) late wandering third instar larvae (*N* = 9–10 per line). Expression is shown relative to the control for each stage. Underlying data can be found in S2 Data. Error bars represent the standard error of the mean. Significance was assessed via a *t* test. *P < 0.05, **P < 0.01, ***P < 0.005.
(PDF)

S7 Fig Density plots of body size and wing loading in a Dutch and Zambian population. Distribution of (A) wing loading, (C) wing area, (E) wing length, and (G) body weight in a Dutch (blue) and a Zambian (yellow) population. Distribution of (B) wing loading, (D) wing area, (F) wing length, and (H) body weight in a Dutch population separated according to the variant at position 67 (6 isofemale lines per variant). The derived, high-expression “G” is shown in blue, and the ancestral, low-expression “C” variant is shown in yellow.
(PDF)

S1 Table Effect of the variant at position 67 on adult reporter gene expression. (PDF)

S2 Table Transcription factor binding site (TFBS) models with predicted differential binding between a representative cosmopolitan and sub-Saharan sequence. (PDF)

S3 Table Site-directed mutagenesis primers. (PDF)

S4 Table Effect of hypomorphic *CG9509*^*del*^ allele on body weight. (PDF)

S5 Table Linkage disequilibrium in the *CG9509* region of a Dutch population. (PDF)

S6 Table Sample sizes for F2 offspring of reciprocal crosses from a Dutch and a Rwandan population. (PDF)

S1 Text Supporting materials and methods. (DOCX)

## Abbreviations

indel: insertion/deletion
qRT-PCR: quantitative reverse transcription PCR
RNAi: RNA interference
SNP: single nucleotide polymorphism
TFBS: transcription factor binding site
UAS: upstream activating sequence
