# Supplementary figures and images for: Functional characterization of adaptive variation within a cis-regulatory element influencing Drosophila melanogaster growth
Source: PLoS Biol. 2018 Jan 11;16(1):e2004538. doi: 10.1371/journal.pbio.2004538 (PMC5783415; doi:10.1371/journal.pbio.2004538)

**A**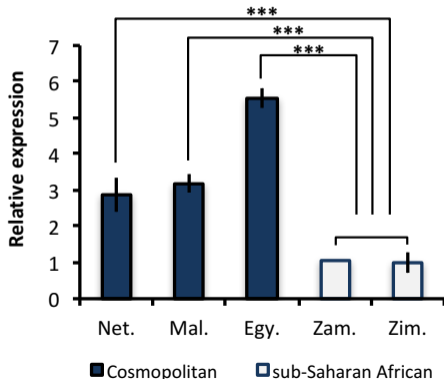**B**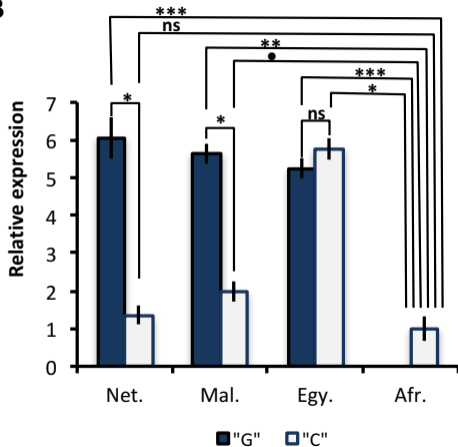

Supplement: S1 Fig — Relative expression in late wandering third instar larvae in (A) the Netherlands (Net.), Malaysia (Mal.), Egypt (Egy.), Zimbabwe (Zim.), and Zambia (Zam.) populations (N = 10–12 isofemale strains per population with 2 biological replicates per strain). Blue bars represent cosmopolitan populations, and white bars represent sub-Saharan populations. (B) Relative expression in each population represented according to the variant at position 67. The high-expression, cosmopolitan “G” variant is shown in blue, and the low-expression, sub-Saharan “C” variant is shown in white. For simplicity, Zambian and Zimbabwean expression are presented together as sub-Saharan African (Afr.) expression. Underlying data can be found in S1 Data. Error bars indicate the standard error of the mean. Differences between populations were tested by a t test, and a Bonferroni multiple test correction was applied. ns, not significant; ●P < 0.10, *P < 0.05, **P < 0.01, ***P < 0.005. (PDF) [file pbio.2004538.s003.pdf]

**A**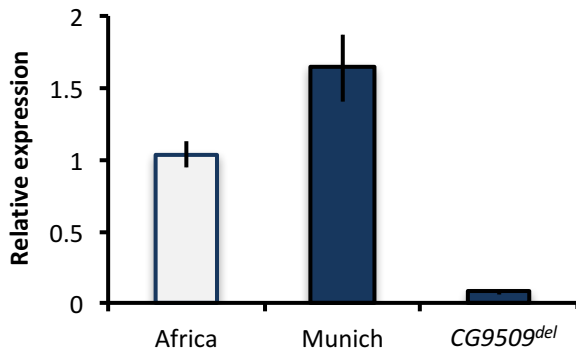**B**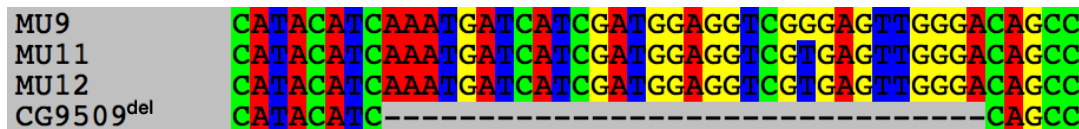**C**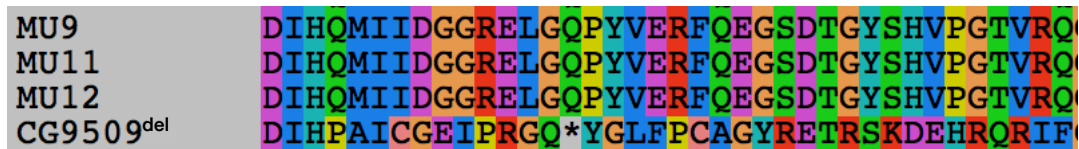

Supplement: S2 Fig — (A) Relative expression of CG9509 in the CG9509del line as determined by quantitative reverse transcription PCR (qRT-PCR). For comparison, the average expression of the population in which the CG9509del line was discovered (Munich) is shown. Underlying data can be found in S1 Data. Error bars represent the standard error of the mean. (B) DNA sequence alignment spanning the deletion within the CG9509del coding region. Sequences of flies from the CG9509del source population in Munich (MU) are shown for comparison. (C) Amino acid alignment spanning the frameshift within the CG9509del coding region. Sequences of flies from the CG9509del source population are shown for comparison (MU). The asterisk indicates a stop codon. (PDF) [file pbio.2004538.s004.pdf]

**A**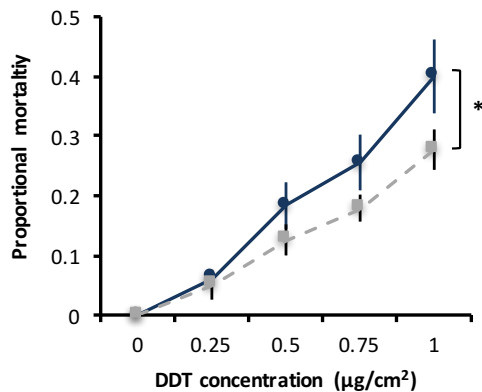**B**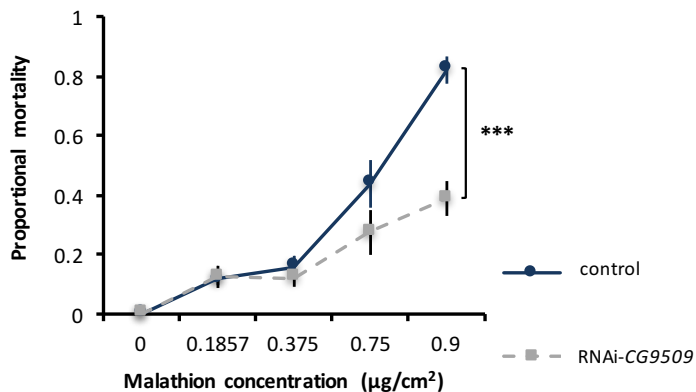**C**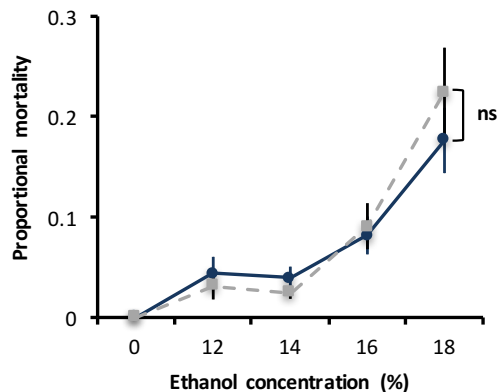**D**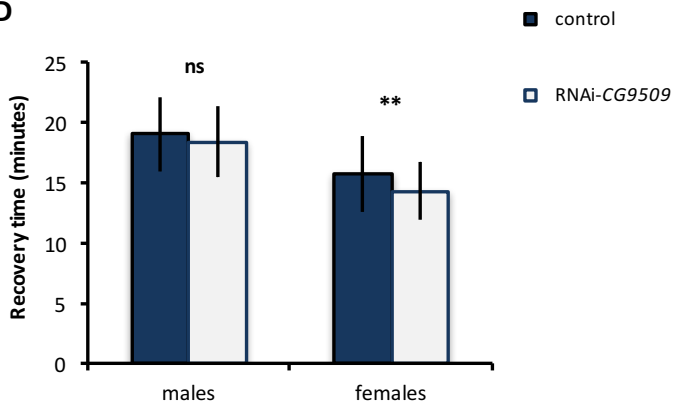

Supplement: S3 Fig — Adult (A) DDT, (B) malathion, (C) ethanol (N = 6–8 replicates per line, sex, and concentration), and (D) cold tolerance assay results (N = 25 per line and sex) for control (blue lines or bars) and RNAi-CG9509 (gray hatched lines or white bars) flies. Underlying data can be found in S2 Data. In panels A–C, significance was assessed using a generalized linear model with a quasibinomial distribution. In panel D, significance was assessed using a t test. ns, not significant; *P < 0.05, **P < 0.01, ***P < 0.005. (PDF) [file pbio.2004538.s005.pdf]

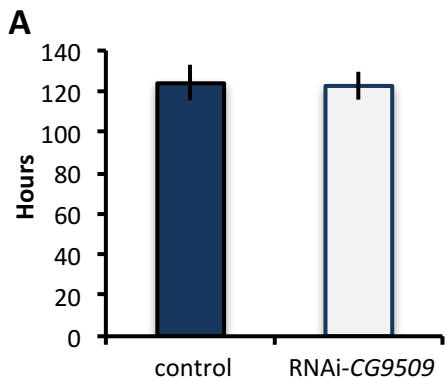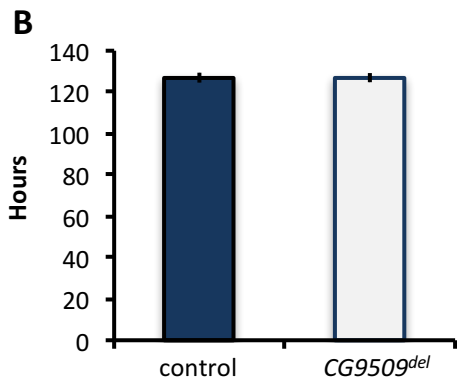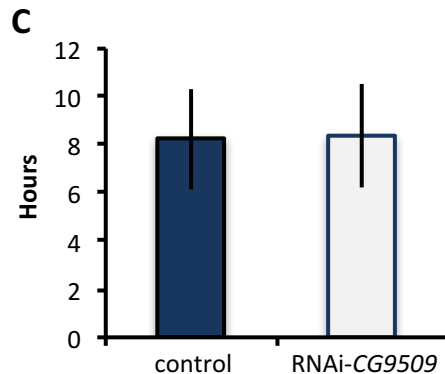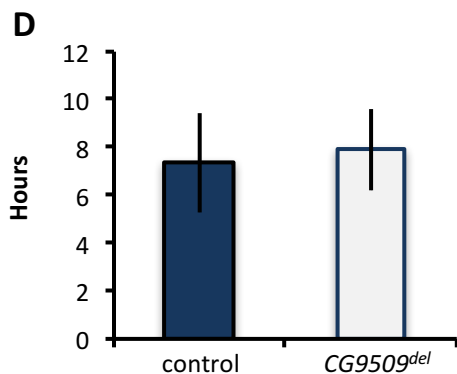

Supplement: S4 Fig — (A, B) Duration of larval stage in (A) control (blue; N = 25) and RNAi-CG9509 (white; N = 51) flies and (B) control (blue; N = 242) and CG9509del (white; N = 107) flies. (C, D) Duration of wandering stage in (C) control (blue; N = 16) and RNAi-CG9509 (white; N = 40) flies and (D) CG9509del (white; N = 10) and control (blue; N = 40) flies. Underlying data can be found in S2 Data. Error bars represent the standard deviation. The knockdown and hypomorph lines were not significantly different from their respective control lines for either stage (t test; P > 0.4 for all comparisons). (PDF) [file pbio.2004538.s006.pdf]

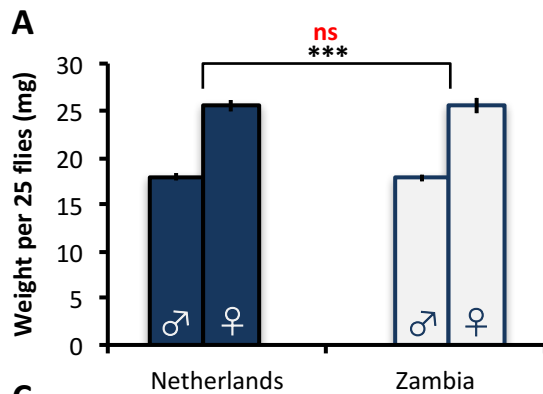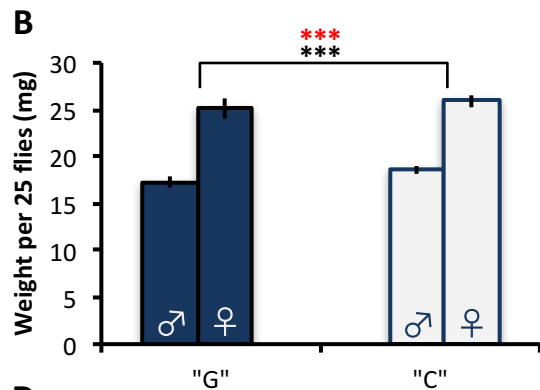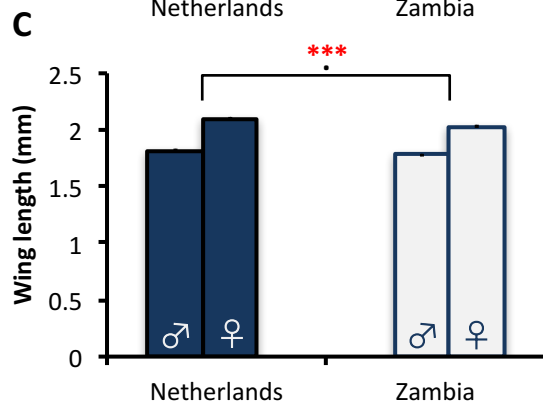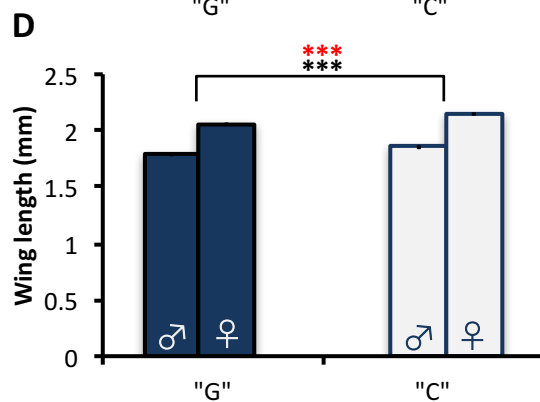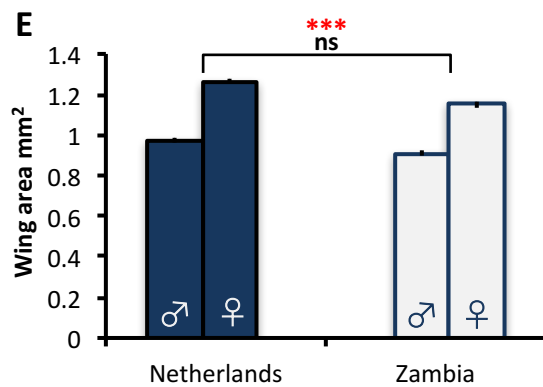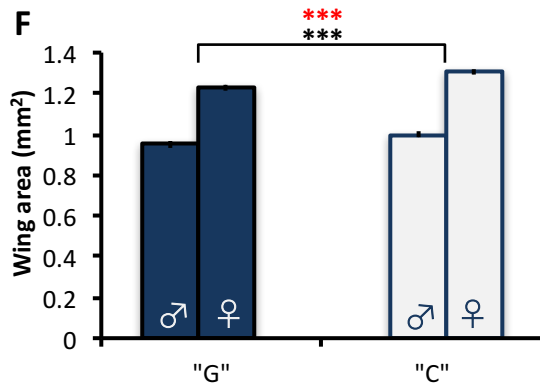

Supplement: S5 Fig — (A) Body weight per 25 flies (N = 10–12 isofemale lines per population with 4 replicates per sex), (C) wing length (N = 10–12 isofemale lines per population with 4 replicates per sex), and (E) wing area in a Dutch (blue bars) and a Zambian (white bars) population (N = 10–12 isofemale lines per population with 4 replicates per sex). (B) Body weight per 25 flies (N = 6 isofemale lines per variant with 4 replicates per sex), (D) wing length (N = 6 isofemale lines per variant with 4 replicates per sex), and (F) wing area in a Dutch population separated according to the variant at position 67 (N = 6 isofemale lines per variant with 4 replicates per sex). The derived, high-expression “G” is shown in blue, and the ancestral, low-expression “C” variant is shown in white. Error bars indicate the standard error of the mean. Underlying data can be found in S2 Data. Significance was assessed with an ANOVA using sex, isofemale line, and population or the variant at position 67 as factors (shown in black). Significance was additionally assessed in both populations simultaneously with population and the variant at position 67 included as factors (shown in red). ns, not significant; ●0.05 < P < 0.10, *P < 0.05, **P < 0.01, ***P < 0.005. (PDF) [file pbio.2004538.s007.pdf]

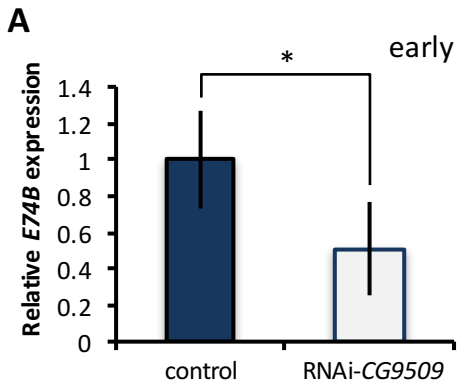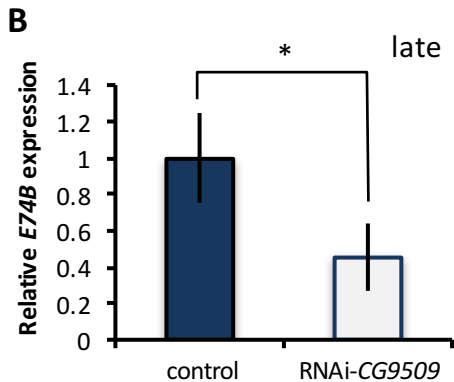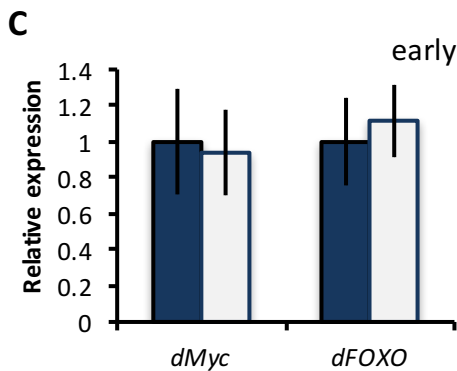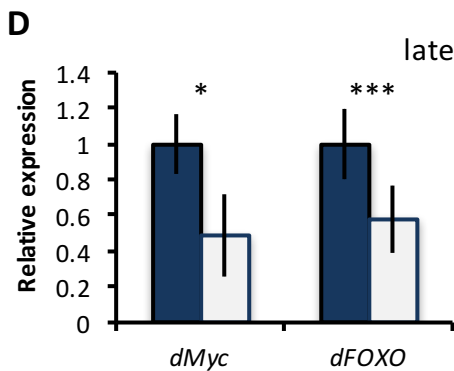

■ control

□ RNAi-CG9509

Supplement: S6 Fig — (A, B) Levels of active ecdysone, approximated by relative E74B expression as measured by quantitative reverse transcription PCR (qRT-PCR) in (A) early and (B) late wandering third instar larvae in control (blue) and RNAi-CG9509 (white) flies (N = 9–10 per line). (C, D) Relative gene expression of dMyc and dFOXO in control (blue) and RNAi-CG9509 (white) (C) early wandering third instar larvae and (D) late wandering third instar larvae (N = 9–10 per line). Expression is shown relative to the control for each stage. Underlying data can be found in S2 Data. Error bars represent the standard error of the mean. Significance was assessed via a t test. *P < 0.05, **P < 0.01, ***P < 0.005. (PDF) [file pbio.2004538.s008.pdf]

**A**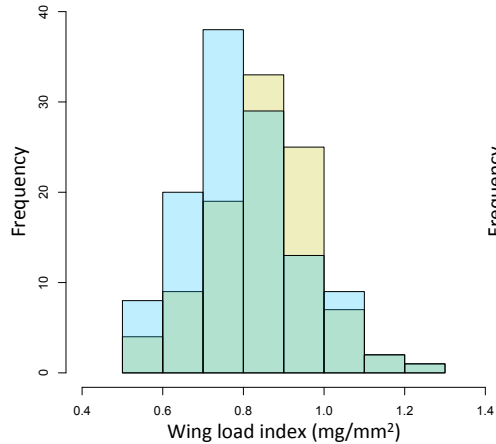**B**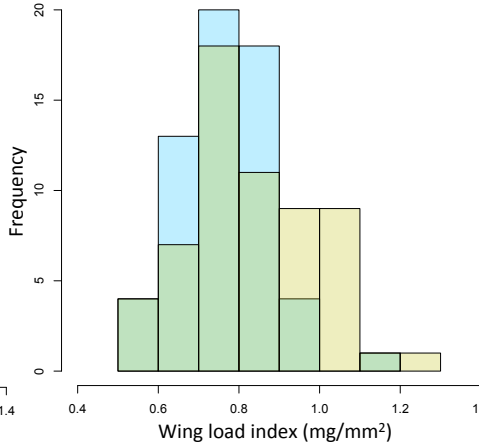**C**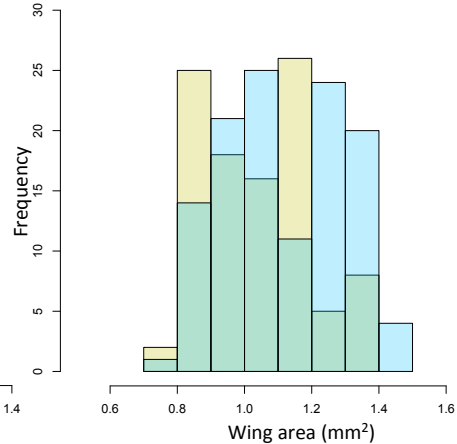**D**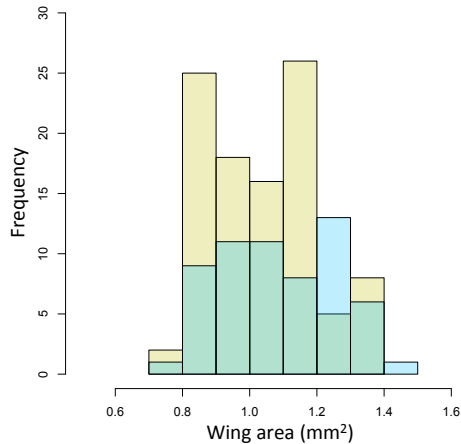**E**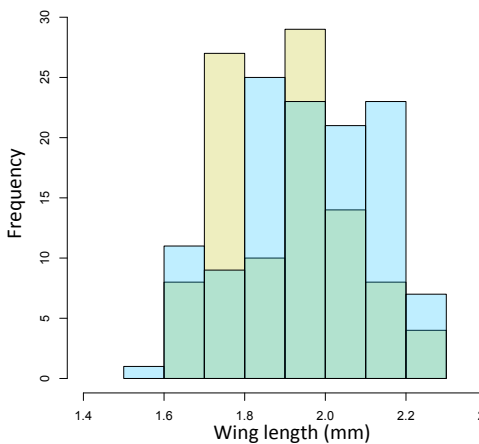**F**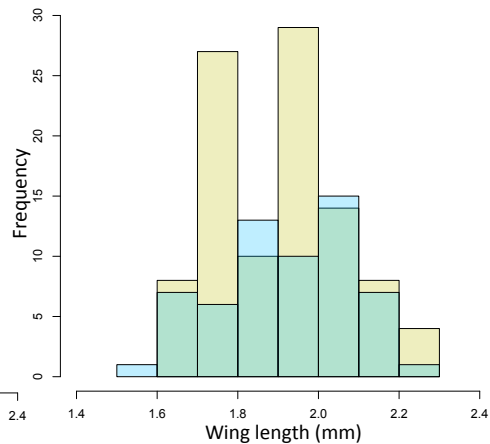**G**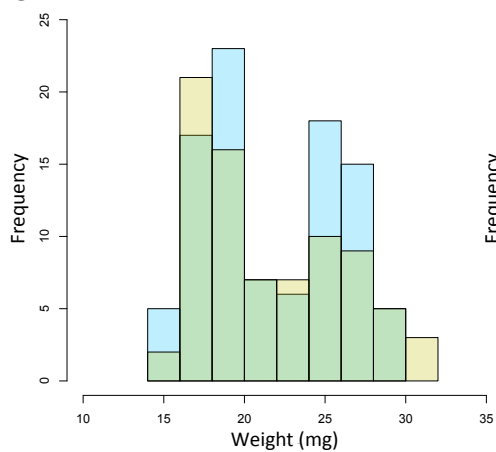**H**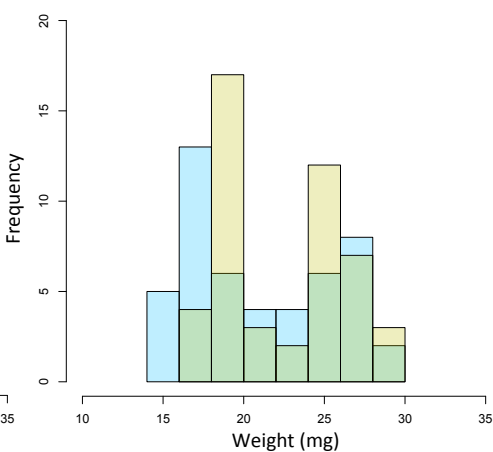

Supplement: S7 Fig — Distribution of (A) wing loading, (C) wing area, (E) wing length, and (G) body weight in a Dutch (blue) and a Zambian (yellow) population. Distribution of (B) wing loading, (D) wing area, (F) wing length, and (H) body weight in a Dutch population separated according to the variant at position 67 (6 isofemale lines per variant). The derived, high-expression “G” is shown in blue, and the ancestral, low-expression “C” variant is shown in yellow. (PDF) [file pbio.2004538.s009.pdf]
