# Supplementary material for: Functional characterization of adaptive variation within a cis-regulatory element influencing Drosophila melanogaster growth
Source: PLoS Biol. 2018 Jan 11;16(1):e2004538. doi: 10.1371/journal.pbio.2004538 (PMC5783415; doi:10.1371/journal.pbio.2004538)
Supplement: S1 Table — (PDF) [file pbio.2004538.s010.pdf]

	η^2_p [a]	SS[b]	DF[c]	F -value[d]	*P*-value[d]
Sex	0.89578	391.7	1	103.1397	3.03E-07
Background	0.77204	154.34	1	40.6407	3.53E-05
SNP67	0.97531	1800.16	1	474.012	5.17E-11
SNP67:Background	0.20285	11.6	1	3.0537	0.10607
SNP67:Sex	0.51954	49.28	1	12.9762	0.003631
SNP67:SNP765	0.55762	57.45	2	7.5631	0.007495
SNP67:SNP115	0.44464	36.49	1	9.6075	0.009197
SNP67:Indel	0.42118	33.16	2	4.3659	0.037606
SNP67:SNP1063	0.12855	6.72	2	0.885	0.437996
SNP67:1174	0.62731	76.71	1	20.1982	0.000734

[a]Effect size of factor on adult reporter gene expression

[b]Sum of Squares

[c]Degrees of freedom

[d]Determined using ANOVA with sex, background, variant at position 67, and the interaction of the variant at position 67 with background, sex, and the other tested positions
