# Supplementary material for: Functional characterization of adaptive variation within a cis-regulatory element influencing Drosophila melanogaster growth
Source: PLoS Biol. 2018 Jan 11;16(1):e2004538. doi: 10.1371/journal.pbio.2004538 (PMC5783415; doi:10.1371/journal.pbio.2004538)
Supplement: S2 Table — (PDF) [file pbio.2004538.s011.pdf]

		Sub-Saharan		Cosmopolitan	
**TF Model[a]**	**SNP**	**Score[b]**	**ln(p-value)[b]**	**Score[b]**	**ln(p-value)[b]**
h	1174	7.37	-9.68	-	-
slbo	1174	3.74	-5.52	-	-
Trl	1174	4.29	-6.36	-	-
twi	1174	4.83	-6.94	-	-
ara	1063	-	-	4.79	-5.45
br(var.3)	1063	-	-	3.58	-5.41
br(var.4)	1063	-	-	1.44	-3.68
caup	1063	-	-	4.18	-5.24
Dll	1063	3.37	-5.2	3.04	-4.93
fkh	1063	-	-	5.13	-7.12
mirr	1063	-	-	5.01	-6.14
slp1	1063	6.41	-8.06	5.8	-7.6
br(var.2)	67	-	-	2.31	-4.13
slbo	67	4.1	-5.87	-	-

[a]Matrix models of predicted transcription factor binding sites were obtained from the JASPAR CORE Insecta database [1].

[b]Score and ln(p-value) were calculated using Patser [2] to survey a representative cosmopolitan and sub-Saharan *CG9509* enhancer sequence (GenBank accession numbers HF913659.1 and HF913717.1).

References

1. Mathelier A, Fornes O, Arenillas DJ, Chen CY, Denay G, Lee J, et al. JASPAR 2016: a major expansion and update of the open-access database of transcription factor binding profiles. Nucleic Acids Res. 2016; 44(D1): D110-5.

2. Hertz GZ, Stormo GD. Identifying DNA and protein patterns with statistically significant alignments of multiple sequences. Bioinformatics. 1999; 15(7-8): 563-77.
