# Supplementary material for: Functional characterization of adaptive variation within a cis-regulatory element influencing Drosophila melanogaster growth
Source: PLoS Biol. 2018 Jan 11;16(1):e2004538. doi: 10.1371/journal.pbio.2004538 (PMC5783415; doi:10.1371/journal.pbio.2004538)
Supplement: S3 Table — (PDF) [file pbio.2004538.s012.pdf]

**Pos.**[a]	**Primer pair (5'->3')**	**Background**[b]	**Overlap**[c]
1174	CGGGCACGCGTTTTAATTACTTTGTAAAGC<br>GTAATTAAAACGCGTGCCCGAAAAGGCGC	Cos.	partial
1174	GGGCAAGCGTTTTAATTACTTTGTTAAGC<br>GTAATTAAAACGCTTGCCCAAAAAGGCGC	Afr.	partial
1155	CGGGCAAGCGTTTTAATTACTTTGTTAAGCTGCATTTTTG<br>CAAAAATGCAGCTTAACAAAGTAATTAAAACGCTTGCCCG	Cos.	full
1063	GCCGTCTTAATGTGTGTTTGTGTCGAGCCAAGTGC<br>CGACACAAACACACATTAAGACGGCAAAAAAAATC	Cos.	partial
1063	CTTAATGTTTGTTTGTGTCGAGCCAAGTGC<br>CGACACAAACAAACATTAAGACAGC	Afr.	partial
765	CAATTTTGTTATTTTTTAAATCTATGCTTTGATTTTAG<br>GCATAGATTTAAAAAATAACAAAATTGTTTTTAAAATTTTATAAC	Cos.	partial
67	CGCGACTGGGCCTCAGAGTCAAATAG<br>CTGAGGC**C**CAGTCGCGGCTGAAGATTCGC	Afr.	partial

[a]Positon (pos.) in bp before *CG9509* start codon

[b]Mutations were introduced into either a Cosmopolitan (Cos.) or sub-Saharan African background (Afr.).

[c]Overlap indicates degree of primer pair overlap.
