# Supplementary material for: Functional characterization of adaptive variation within a cis-regulatory element influencing Drosophila melanogaster growth
Source: PLoS Biol. 2018 Jan 11;16(1):e2004538. doi: 10.1371/journal.pbio.2004538 (PMC5783415; doi:10.1371/journal.pbio.2004538)
Supplement: S4 Table — (PDF) [file pbio.2004538.s013.pdf]

**Genotype**[a]	**Sex**	**N**	**Weight**[b] **(mg) ± SEM**
H/H	female	14	1.6743 ± 0.0401
H/WT	female	22	1.6700 ± 0.0418
WT/WT	female	10	1.5940 ± 0.0551
H	male	25	1.3652 ± 0.0233
WT	male	19	1.2937 ± 0.0285

[a] *CG9509* genotype in F2 offspring of a cross between flies carrying a hypomorph allele (H) or a wild-type allele (WT). Males are hemizygous and carry only one copy.

[b] Effect of *CG9509* allele on adult body weight: $P = 0.0373$, $\eta^2_p = 0.049$. Significance assessed using an ANOVA with allele and sex as factors.
