# Supplementary material for: Functional characterization of adaptive variation within a cis-regulatory element influencing Drosophila melanogaster growth
Source: PLoS Biol. 2018 Jan 11;16(1):e2004538. doi: 10.1371/journal.pbio.2004538 (PMC5783415; doi:10.1371/journal.pbio.2004538)
Supplement: S5 Table — (PDF) [file pbio.2004538.s014.pdf]

Pos.[a]	Dist.[b]	r^{2c}	*P*-value[d]	Pos.[a]	Dist.[b]	r^{2c}	*P*-value[d]
26082	24969	0.312481	0.182	35838	15213	0.001849	1
26323	24728	0.266256	0.182	35955	15096	0.001849	1
37940	13111	0.312481	0.182	36050	15001	0.01	1
39435	11616	0.218089	0.242	37781	13270	0.01	1
39667	11384	0.218089	0.242	38183	12868	0.01	1
39680	11371	0.201601	0.242	38206	12845	0.01	1
39697	11354	0.218089	0.242	38335	12716	0.01	1
39720	11331	0.218089	0.242	38444	12607	0.01	1
39835	11216	0.218089	0.242	38728	12323	0.001849	1
40429	10622	0.201601	0.242	38998	12053	0.004761	1
41594	9457	0.218089	0.242	39307	11744	0.022201	1
41811	9240	0.218089	0.242	39311	11740	0.001849	1
42545	8506	0.218089	0.242	40110	10941	0.001849	1
42730	8321	0.201601	0.242	40351	10700	0.001849	1
42893	8158	0.201601	0.242	40361	10690	0.001849	1
43817	7234	0.218089	0.242	40362	10689	0.001849	1
43867	7184	0.218089	0.242	40376	10675	0.001849	1
43885	7166	0.218089	0.242	40967	10084	0.001849	1
43953	7098	0.218089	0.242	41431	9620	0.001849	1
44130	6921	0.201601	0.242	42828	8223	0.022201	1
44193	6858	0.201601	0.242	42874	8177	0.022201	1
44333	6718	0.201601	0.242	43215	7836	0.001849	1
44676	6375	0.201601	0.242	44993	6058	0.001849	1
46488	4563	0.201601	0.242	45133	5918	0.001849	1
47759	3292	0.201601	0.242	45153	5898	0.001849	1
47780	3271	0.201601	0.242	45221	5830	0.001849	1
26399	24652	0.1849	0.455	45242	5809	0.001849	1
26475	24576	0.1849	0.455	45244	5807	0.001849	1
26478	24573	0.1849	0.455	45354	5697	0.001849	1
26480	24571	0.1849	0.455	45355	5696	0.001849	1
26500	24551	0.1849	0.455	45402	5649	0.001849	1
26544	24507	0.1849	0.455	45587	5464	0.004761	1
26581	24470	0.1849	0.455	45809	5242	0.01	1
26582	24469	0.1849	0.455	45904	5147	0.004761	1
29279	21772	0.1849	0.455	45905	5146	0.004761	1
37921	13130	0.1849	0.455	45931	5120	0.004761	1
38537	12514	0.1849	0.455	45937	5114	0.004761	1
38575	12476	0.1849	0.455	45963	5088	0.004761	1
39178	11873	0.1849	0.455	46269	4782	0.004761	1
41194	9857	0.1849	0.455	46272	4779	0.004761	1
41269	9782	0.1849	0.455	46314	4737	0.004761	1
41297	9754	0.1849	0.455	48021	3030	0.022201	1

41304	9747	0.1849	0.455
41369	9682	0.1849	0.455
41378	9673	0.1849	0.455
41381	9670	0.1849	0.455
42178	8873	0.1849	0.455
49329	1722	0.1849	0.455
27248	23803	0.096721	0.545
27259	23792	0.096721	0.545
27400	23651	0.096721	0.545
27405	23646	0.096721	0.545
27452	23599	0.096721	0.545
27459	23592	0.096721	0.545
27486	23565	0.096721	0.545
27511	23540	0.096721	0.545
27586	23465	0.096721	0.545
27707	23344	0.096721	0.545
27944	23107	0.096721	0.545
28105	22946	0.096721	0.545
28494	22557	0.096721	0.545
28802	22249	0.096721	0.545
28916	22135	0.096721	0.545
29135	21916	0.096721	0.545
29516	21535	0.096721	0.545
40711	10340	0.096721	0.545
41685	9366	0.096721	0.545
42107	8944	0.096721	0.545
42124	8927	0.096721	0.545
42637	8414	0.096721	0.545
42969	8082	0.096721	0.545
42980	8071	0.096721	0.545
42984	8067	0.096721	0.545
43017	8034	0.096721	0.545
43136	7915	0.096721	0.545
49167	1884	0.096721	0.545
40689	10362	0.071289	0.567
40690	10361	0.071289	0.567
40975	10076	0.071289	0.567
41316	9735	0.071289	0.567
41317	9734	0.071289	0.567
44514	6537	0.071289	0.567
46029	5022	0.071289	0.567
46095	4956	0.071289	0.567
27608	23443	0.022201	1
29759	21292	0.022201	1

49375	1676	0.01	1
49485	1566	0.022201	1
49617	1434	0.01	1
50016	1035	0.01	1
50046	1005	0.01	1
50195	856	0.01	1
50347	704	0.001849	1
50612	439	0.001849	1
52971	1920	0.001849	1
53590	2539	0.266256	0.182
53703	2652	0.266256	0.182
53741	2690	0.266256	0.182
53764	2713	0.266256	0.182
54066	3015	0.004761	1
54198	3147	0.068121	0.545
54222	3171	0.266256	0.182
54300	3249	0.266256	0.182
54444	3393	0.068121	0.545
54501	3450	0.266256	0.182
54504	3453	0.266256	0.182
54837	3786	0.071289	0.567
54843	3792	0.004761	1
54906	3855	0.266256	0.182
54966	3915	0.266256	0.182
54969	3918	0.266256	0.182
55035	3984	0.266256	0.182
55092	4041	0.004761	1
55193	4142	0.266256	0.182
55643	4592	0.096721	0.545
55670	4619	0.218089	0.242
55682	4631	0.201601	0.242
55854	4803	0.312481	0.182
55878	4827	0.312481	0.182
55941	4890	0.4761	0.061
55955	4904	0.4761	0.061
55962	4911	0.4761	0.061
55992	4941	0.4761	0.061
55997	4946	0.4761	0.061
56012	4961	0.312481	0.182
56081	5030	0.312481	0.182
56146	5095	0.4761	0.061
56258	5207	0.201601	0.242
56445	5394	0.450241	0.061
58880	7829	0.266256	0.182

29795	21256	0.022201	1	59077	8026	0.001849	1
30033	21018	0.001849	1	59327	8276	0.068121	0.545
30088	20963	0.022201	1	59366	8315	0.004761	1
30155	20896	0.022201	1	59624	8573	0.001849	1
30159	20892	0.022201	1	59648	8597	0.001849	1
30194	20857	0.022201	1	59924	8873	0.096721	0.545
30224	20827	0.022201	1	59927	8876	0.096721	0.545
30359	20692	0.022201	1	60083	9032	0.1849	0.455
30828	20223	0.022201	1	60098	9047	0.1849	0.455
31511	19540	0.004761	1	60171	9120	0.1849	0.455
31585	19466	0.004761	1	60185	9134	0.1849	0.455
31811	19240	0.01	1	60885	9834	0.001849	1
32248	18803	0.01	1	63896	12845	0.1849	0.455
32250	18801	0.01	1	65500	14449	0.071289	0.567
32590	18461	0.001849	1	65533	14482	0.1849	0.455
32713	18338	0.01	1	65563	14512	0.001849	1
32981	18070	0.001849	1	65837	14786	0.001849	1
32988	18063	0.001849	1	65933	14882	0.001849	1
33303	17748	0.022201	1	66018	14967	0.1849	0.455
33443	17608	0.022201	1	66829	15778	0.001849	1
33729	17322	0.001849	1	68040	16989	0.266256	0.182
33735	17316	0.001849	1	68063	17012	0.266256	0.182
33737	17314	0.001849	1	74995	23944	0.004761	1
33774	17277	0.001849	1	75115	24064	0.1849	0.455
34564	16487	0.01	1	75146	24095	0.1849	0.455
35029	16022	0.01	1	75161	24110	0.1849	0.455
35051	16000	0.01	1	75186	24135	0.1849	0.455
35301	15750	0.001849	1	75315	24264	0.096721	0.545
35817	15234	0.001849	1	75713	24662	0.096721	0.545

[a]Position (pos.) in alignment. Dutch full genome sequences are described in [1] and available from http://evol.bio.lmu.de/downloads/.
[b]Distance (dist.) from position 67 in *CG9509* enhancer in base pairs
[c]The degree of linkage disequilibrium, r^2 [2], between position 67 and the indicated position. All polymorphic sites, excluding singletons, within a 50 kb region centered around position 67 are listed.

References

1. Voigt S, Laurent S, Litovchenko M, Stephan W. Positive Selection at the *Polyhomeotic* Locus Led to Decreased Thermosensitivity of Gene Expression in Temperate *Drosophila melanogaster*. Genetics. 2015; 200(2): 591-9.

2. Lewontin RC. The interaction of selection and linkage. II. Optimum models. Genetics. 1964; 50: 757-82.
