# Supplementary material for: Functional characterization of adaptive variation within a cis-regulatory element influencing Drosophila melanogaster growth
Source: PLoS Biol. 2018 Jan 11;16(1):e2004538. doi: 10.1371/journal.pbio.2004538 (PMC5783415; doi:10.1371/journal.pbio.2004538)
Supplement: S6 Table — (PDF) [file pbio.2004538.s015.pdf]

Pos.[a]	Genotype[b]	Sex[c]	N[d]	N_{wing}[e]
67	G/G	F	13	11
67	G/C	F	24	24
67	C/C	F	13	13
67	G	M	27	24
67	C	M	23	21
1174 & 1063	AT/AT	F	29	29
1174 & 1063	CG/CG	F	35	35
1174 & 1063	AT	M	28	27
1174 & 1063	CG	M	34	32

[a]Position (pos.) of interest in *CG9509* enhancer

[b]Genotype at position of interest

[c]Male (M) or female (F)

[d]Number of flies for which body weight was measured

[e]Number of flies for which wing size and wing loading was measured
