# Supplementary material for: Functional characterization of adaptive variation within a cis-regulatory element influencing Drosophila melanogaster growth
Source: PLoS Biol. 2018 Jan 11;16(1):e2004538. doi: 10.1371/journal.pbio.2004538 (PMC5783415; doi:10.1371/journal.pbio.2004538)
Supplement: S1 Text — (DOCX) [file pbio.2004538.s016.docx]

**S1 Text. Supporting Materials and Methods**

***D. melanogaster* lines**

**Association between *CG9509^del^* line and body weight.** Although we used four control lines for all analyses of *CG9509^del^* phenotypes and these results were consistent with those seen in *CG9509* RNAi-knockdown flies (Figs 4­–7, S3, S6), it remains possible the observed *CG9509^del^* phenotypes are caused by variation in another part of the genome of the *CG9509^del^* line. Therefore, we confirmed that the observed phenotypes are associated with the *CG9509^del^* allele by performing reciprocal crosses between the *CG9509^del^* line and a control line and measured adult body weight in reconstituted homozygotes, heterozygotes, and hemizygotes in the F2 generation. Reciprocal crosses of 30–40 females and 15–20 males were performed, 40–50 F1 progeny were allowed to mate randomly, and adult body weight was measured in the F2 generation. After weighing, flies were stored at -80°C in 96% ethanol before extraction of DNA from either the head or a leg using the Animal Direct PCR kit (Thermo Fisher Scientific; Waltham, Massachusetts, USA). For genotyping, a ~1-kb fragment was amplified using the following primers (5’–3’): CTGAGCGAGAATCCCGACTG and GGTCACTTTCACTCGATGGGT, followed by digestion with *Cla*I, which cuts the wild-type but not the hypomorph allele into ~800 and ~250 bp fragments. Presence of the hypomorph allele significantly increased weight (*P* = 0.0373, S4 Table). Significance was assessed using an ANOVA with sex and allele as factors. The effect of the allele was assumed to be additive, with hypomorph homo- and hemizygotes assigned a value of 2, wild-type homo- and hemizygotes assigned a value of 0, and heterozygotes assigned a value of 1.

**Crosses to test association between *CG9509* enhancer SNPs and phenotype.** After collection of phenotype data, flies were stored at -80°C in 96% ethanol before extraction of DNA from either the head or a leg using the Animal Direct PCR kit (Thermo Fisher Scientific; Waltham, Massachusetts, USA). The *CG9509* enhancer sequences for the Dutch lines used in crosses were previously sequenced [1] and are available on GenBank (Accession numbers: HF913659.1 and HF913664.1). For genotyping, a ~1.4-kb fragment around the SNP at position 67 was amplified using the following primers (5’­–3’): TCGTGATGATGCCGATTCAGA and CCCGATCCTCCCAGCATTTT, followed by digestion with *Hha*I, which cuts the “C” but not the “G” variant into two fragments of ~925 and ~450 bp. The *CG9509* enhancer sequences for the Rwandan lines (RG11, RG25, and RG28) used in crosses were amplified as described in [2], sequenced using the primers described in the transgenic reporter gene constructs below, and deposited in GenBank under the accession numbers: MG195568 – MG195570. RG25 and RG11 contain either cosmopolitan (RG25) or sub-Saharan (RG11) variants at positions 1174, 1155, 1063, and 821–817, but are identical at other sites of interest in the *CG9509* enhancer. RG28 contains cosmopolitan variants at these positions as well as at positions 167 and 67, while RG11, contains sub-Saharan variants at these positions. For genotyping, flies were sequenced as described above and for the crosses between RG28 and RG11 only offspring containing sub-Saharan African variants at positions 167 and 67 were used. Sequences for all parental lines used in crosses were verified by independently sequencing 3–5 flies per line.

**Recombination in Rwandan F2 offspring.** In the F2 generation of a pair of reciprocal crosses between two homozygous isofemale strains of *D. melanogaster*, ~50% of all female offspring are expected to be heterozygous at an X-linked locus. For both pairs of Rwandan crosses, we recovered only females that were homozygous at the *CG9509* locus, which is significantly different from the expectation (Haldane Exact test, *P* < 10^-15^). These results suggest that i) heterozygotes at this (or a linked) locus are lethal, ii) heterozygotes at this (or a linked) locus have a significantly different developmental time than homozygotes, iii) there are incompatibilities between these lines, or iv) some type of meiotic drive is occurring. While i) and ii) are unlikely as the *CG9509^del^* allele is not lethal and changes in *CG9509* expression do not affect developmental time (S4 Fig), we cannot completely rule out this possibility for closely linked loci. Nevertheless, none of these causes should affect our results as long as enough recombination has occurred to separate the *CG9509* enhancer from other potentially linked sites.

The reciprocal crosses between RG28 and RG11 provide an internal check for determining if recombination can sufficiently break up loci closely linked to the *CG9509* gene region within two generations of full sibling mating, as a recombination event is required to occur within the *CG9509* enhancer itself in order to obtain cosmopolitan variants at positions 1174, 1155, 1063, and 821–817 in the desired background. While RG11 contains sub-Saharan variants and RG28 contains cosmopolitan variants at positions 1174, 1155, 1063, and 821–817, they also differ at positions 167 and 67, where RG28 contains cosmopolitan variants while RG11 contains sub-Saharan variants. Thus, recombination must occur in order to generate F2 offspring with cosmopolitan variants at positions 1174, 1155, 1063, and 821–817 but otherwise identical to RG11 at other sites of interest in the *CG9509* enhancer. Of the 52 F2 offspring in this study from crosses between RG11 and RG28, 5 contained cosmopolitan variants at positions 1174, 1155, 1063, and 821–817 but were otherwise identical to RG11 at other sites of interest in the *CG9509* enhancer, and thus represent recombination events that occurred within the *CG9509* enhancer itself. Thus, recombination was efficient enough to break up even closely linked variants in these crosses.

**Linkage disequilibrium analysis**

In the Dutch population we calculated the degree of linkage disequilibrium, *r^2^* [3], between position 67 and all SNPs, excluding singletons, within a 50-kb region centered around position 67 using DnaSP (version 5) [4] and significance was assessed with a Fisher’s Exact test. Analysis was performed using the Dutch full genome sequences described in [5] and available from http://evol.bio.lmu.de/downloads/.

**Construction of transgenic reporter gene constructs**

The effects of six sub-Saharan sequence variants (positions 67, 765, 821–817, 1063, 1155, and 1174; Fig 2B) in the cosmopolitan background were examined. Because the sub-Saharan variant “C” at position 67 is polymorphic in cosmopolitan populations, the enhancer region was PCR-amplified from a cosmopolitan strain straining containing a “C” variant at position 67 and cloned into the *TOPO* vector as described in the main text. Due to the complexity of the region in which the 5-bp indel at 822–817 bp is located, a synthesized vector containing parts of the *TOPO* vector, the cosmopolitan upstream variant, and the desired 5 bp insertion was custom ordered from GeneArt (Invitrogen; Carlsbad, California, USA). A 540 bp *Spe*I/*Nde*I fragment of this vector was then ligated into the *TOPO* vector containing the cosmopolitan enhancer sequence.

The four other sub-Saharan African variants were introduced into the cosmopolitan sequence via site-directed mutagenesis, which was performed using thermal cycling with PfuUltra II Fusion HS DNA polymerase (Agilent Technologies; Santa Clara, California, USA) and complimentary, fully or partially overlapping primer pairs containing the desired base pair change [6] (for primer list see S3 Table). For sites that were shown to affect reporter gene expression, the cosmopolitan variants were introduced into the sub-Saharan enhancer and constructs with all contributing sites were generated in both a cosmopolitan and sub-Saharan background using site-directed mutagenesis as described above for a total of 13 reporter gene constructs. The original and the mutated enhancer sequences were confirmed via sequencing using the following primers (5’–3’): TTTGGTTTCCTTACCGTTTTG, GTGCAGTTTGGAACTCAG, CATTTATAGCACTTGGCTCG, GCTTCGCATTCTGGATGC, TGGCGCTAACCTGAATTCC, and GCGTTTTGCTTTTCCGTTAG. A 3.5-kb *Not*I fragment of the *pCMV-SPORT-βgal* plasmid (Invitrogen; Carlsbad, California, USA) containing the *E. coli LacZ* coding region was then inserted into the *Not*I site located just downstream of the enhancer sequence in the *TOPO* vector and a *Bam*HI/*Xba*I fragment containing both the *CG9509* enhancer and *LacZ* sequences was subsequently excised and ligated into the *pattB* integration vector [7].

**Larval staging**

In order to control for size and age, all larval staging was performed at 25°C with a 14 hr light:10 hr dark cycle. Adult females were placed in cages and allowed to lay eggs on molasses-agar plates supplemented with yeast for 48 hours for expression analyses and β-galactosidase assays or for 4 hours for larval growth rate analysis. First instar larvae (L1) were then collected for staging. To stage second instar larvae (L2) or early third instar larvae (L3), first instar larvae were transferred to small molasses-agar plates supplemented with cornmeal-molasses medium at a density of 50 larvae per plate. Second instar larvae were collected ~48 hours after egg laying (shortly after the L1-L2 transition) and identified by their mouth hooks. Early third instar larvae were collected ~72 hours after egg laying (shortly after the L2-L3 transition) and were identified by their spiracles. To stage wandering third instar larvae, first instar larvae were transferred to large vials containing cornmeal-molasses medium supplemented with 5% bromophenol blue (Carl Roth; Karlsruhe, Germany) at a density of 150 larvae per vial. Early third instar wandering larvae (shortly after onset of wandering, puff stages 1-2) were identified by the dark blue color of their guts, while late third instar wandering larvae (shortly before pupariation, puff stages 7–9) were identified by their clear guts. Larvae were washed in PBS and gently dried then kept on ice until use. Larvae for RNA extraction were stored at -80°C before use.

**Adult staging**

Since temperature and density during rearing as well as age can affect body size and wing loading [8–10], the flies used in body size and wing loading assays were strictly controlled. All staging took place at 25°C. Adults were placed in cages and allowed to lay eggs on molasses-agar plates supplemented with yeast for 48 hours. First instar larvae were collected and placed in vials at a density of 50 larvae per vial. After eclosion, flies were collected and sorted into groups of males and females every 3–4 hours, so that only virgin flies were used. Flies were aged for one day before use in assays.

**References**

1. Glaser-Schmitt A, Catalán A, Parsch J. Adaptive divergence of a transcriptional enhancer between populations of *Drosophila melanogaster*. Philos Trans R Soc Lond B Biol Sci. 2013; 368(1632): 20130024.

2. Saminadin-Peter SS, Kemkemer C, Pavlidis P, Parsch J. Selective sweep of a *cis*-regulatory sequence in a non-African population of *Drosophila melanogaster*. Mol Biol Evol. 2012; 29(4): 1167-74.

3. Lewontin RC. The interaction of selection and linkage. II. Optimum models. Genetics. 1964; 50: 757-82.

4. Librado P, Rozas J. DnaSP v5: a software for comprehensive analysis of DNA polymorphism data. Bioinformatics. 2009; 25(11): 1451-2.

5. Voigt S, Laurent S, Litovchenko M, Stephan W. Positive Selection at the *Polyhomeotic* Locus Led to Decreased Thermosensitivity of Gene Expression in Temperate *Drosophila melanogaster*. Genetics. 2015; 200(2): 591-9.

6. Zheng L, Baumann U, Reymond J-L. An efficient one-step site-directed and site-saturation mutagenesis protocol. Nucleic Acids Res. 2004; 32(14): e115-e.

7. Bischof J, Maeda RK, Hediger M, Karch F, Basler K. An optimized transgenesis system for *Drosophila* using germ-line-specific φC31 integrases. Proc Natl Acad Sci U S A. 2007; 104(9): 3312-7.

8. James AC, Partridge L. Thermal evolution of rate of larval development in *Drosophila melanogaster* in laboratory and field populations. J Evol Biol. 1995; 8(3): 315-30.

9. Santos M, Fowler K, Partridge L. Gene-environment interaction for body size and larval density in *Drosophila melanogaster*: an investigation of effects on development time, thorax length and adult sex ratio. Heredity. 1994; 72(5): 515-21.

10. Angilletta MJ, Steury TD, Sears MW. Temperature, growth rate, and body size in ectotherms: fitting pieces of a life-history puzzle. Integr Comp Biol. 2004; 44(6): 498-509.
